# Mechanisms of Anti-Aging Effect of *Alpinia oxyphylla* Polysaccharides Mediated via IIS Pathway: Based on In Vivo Experiments, Network Pharmacology and Molecular Docking

**DOI:** 10.3390/molecules31101698

**Published:** 2026-05-17

**Authors:** Taixia Chen, Yan Wang, Yilong Wu, Kaibo Feng, Qiuling Wang, Yiquan Lan, Qiangqiang Zhu, Xiaoyun Wu, Jun Sheng, Chengting Zi

**Affiliations:** 1 College of Food Science and Technology, Yunnan Agricultural University, Kunming 650201, China; 15870148875@163.com (T.C.); 17386583693@163.com (K.F.); qiulingwang2022@163.com (Q.W.); tianjiao125@126.com (Q.Z.); 2 Key Laboratory of Development and Utilization of Food and Medicinal Resources, Yunnan Agricultural University, Kunming 650201, China; 3 Institute of Biofabrication Research, College of Science, Yunnan Agricultural University, Kunming 650201, China; wangyan2882@163.com (Y.W.); ynau_wuyl@126.com (Y.W.); lanyiquan2025@163.com (Y.L.); 4 Research Center for Agricultural Chemistry, College of Science, Yunnan Agricultural University, Kunming 650201, China

**Keywords:** *Alpinia oxyphylla* polysaccharides, anti-aging, *Caenorhabditis elegans*, IIS pathway, network pharmacology, molecular docking

## Abstract

Background: This study aimed to investigate the anti-aging mechanisms of *Alpinia oxyphylla* polysaccharides (AOFs) through integrated in vivo experiments, network pharmacology, and molecular docking. Methods: Three purified fractions (AOF1, AOF2, and AOF3) were structurally characterized for monosaccharide composition and molecular weight. Anti-aging and antioxidant activities were evaluated using *Caenorhabditis elegans*, followed by gene expression analysis, network pharmacology target identification, and molecular docking validation. Results: All AOFs significantly extended lifespan, enhanced resistance to oxidative and heat stress, reduced reactive oxygen species and lipid peroxidation, and upregulated superoxide dismutase and catalase activities. Gene expression analysis revealed activation of the insulin/insulin-like growth factor signaling pathway through upregulation of daf 16, skn 1, sod 3, ctl 1, and hsp 16.2. Network pharmacology identified 254, 85, and 119 core targets for AOF1, AOF2, and AOF3 respectively, enriched in PI3K/AKT, MAPK, hypoxia, and xenobiotic response pathways. KEGG analysis further implicated lipid and atherosclerosis, HIF 1, FoxO, and PI3K Akt signaling. Molecular docking showed that critical monosaccharides and metformin formed stable hydrogen-bonded complexes with AKT1, INS, SRC, and STAT3. Among the fractions, AOF1 and AOF3 exhibited superior activities. Conclusions: These findings demonstrate the multi-target, multi-pathway anti-aging actions of AOFs and support their potential as natural antioxidants and functional food ingredients for anti-aging therapeutics.

## 1. Introduction

The unprecedented global aging trend presents critical societal challenges to sustaining quality of life and extending healthy life expectancy, thereby placing increasing burdens on already strained healthcare systems [1]. Aging is a complex and multifactorial biological process involving telomere attrition, DNA damage, mitochondrial dysfunction, chronic inflammation, and other cellular deteriorations [2]. This gradual functional decline is frequently accompanied by the onset of chronic diseases, including cardiovascular and cerebrovascular disorders, degenerative joint diseases, diabetes, Parkinson’s disease, Alzheimer’s disease, and various cancers. Consequently, identifying safe and effective strategies to regulate health status and extend the healthy lifespan of the elderly population has become a global priority in promoting healthy aging.

In response to these challenges, multiple interventions have been proposed to mitigate age-related decline. These strategies cover preventive healthcare measures focusing on balanced nutrition and regular physical exercise [3] and balanced dietary patterns dominated by plant-based foods and high-quality animal products [4,5], as well as the research and development of anti-aging drugs and therapeutic agents [6,7,8]. However, given the extreme complexity of aging mechanisms, no standardized and universally effective anti-aging intervention has been developed to date. Previous research has highlighted the urgent need to develop novel drug candidates that delay aging and extend lifespan [9]. At present, widely used anti-aging agents include senolytics, metformin, resveratrol, rapamycin, sedatives, and certain antibiotics [10,11,12,13]. These compounds exert anti-aging effects via multiple biological pathways, including improving mitochondrial function, restoring stem cell activity, regulating epigenetic modifications, inducing autophagy, and alleviating DNA damage and telomere shortening [14]. Nevertheless, their efficacy remains inadequately supported by clinical data, and their potential adverse effects warrant careful consideration [15]. Hence, there is an urgent need to explore natural, safe, and sustainable alternatives for anti-aging drug discovery.

Polysaccharides are natural macromolecular polymers formed through condensation and dehydration reactions. Derived from renewable biomass resources, plant polysaccharides are environmentally friendly and sustainable biopolymers that are widely distributed in plant cell walls and membranes. They exhibit diverse biological activities including anti-inflammatory, antitumor, antioxidant, anti-aging, and immunomodulatory effects while offering advantages such as low toxicity, safety, and minimal residue [9,16,17,18]. Emerging evidence suggests that plant polysaccharides play pivotal roles in scavenging free radicals, regulating telomerase activity, inhibiting apoptosis, enhancing immune responses, and modulating neuroendocrine functions. These processes are believed to influence key signal transduction pathways involved in aging, highlighting the potential of polysaccharides as bioactive regulators of longevity [19]. Researchers found that *Ganoderma* lucidum polysaccharides can delay aging and enhance anti stress ability by regulating IIS and MAPK pathways in *C. elegans* [20]. O-acetyl glucomannan (LPR) mainly provides heat resistance and antioxidant capacity by enhancing the function of the antioxidant defense system of nematodes and scavenging free radicals, and prolongs the life of wild-type nematodes [21]. Indeed, numerous plant-derived polysaccharides have demonstrated potent anti-aging effects across multiple model organisms, stimulating growing scientific interest in their therapeutic application.

*Alpinia oxyphylla* (AO), a traditional medicinal and edible plant primarily cultivated in Fujian, Guangdong, and Hainan, is recognized as one of the four major herbal medicines of southern China. It possesses a broad spectrum of pharmacological properties, including anti-cancer, anti-inflammatory, antioxidant, anti-aging, neuroprotective, immunomodulatory, and protective effects on the cardiovascular and gastrointestinal systems [22,23,24]. Polysaccharides isolated from *A. oxyphylla* (AOP) have been extracted using ultrasound-assisted enzymatic extraction [25], hot-water extraction [26], optimized extraction via response surface methodology [27], and aqueous alcoholic precipitation [28]. The principal monosaccharides identified in *A. oxyphylla* polysaccharides include arabinose, galactose, glucose, xylose, mannose, galacturonic acid, and glucuronic acid, with glucose consistently detected across samples [28]. Polysaccharides extracted from *A. oxyphylla* (AOFs) have been reported to exhibit significant anti-inflammatory, antioxidant, and immunomodulatory activities [25,29]. Despite these promising findings, the anti-aging potential of AOFs and their underlying mechanisms remain largely unexplored. Given that the biological activities of plant polysaccharides are profoundly influenced by their structural characteristics and purity, comprehensive mechanistic investigations are essential. Moreover, systematic studies elucidating the pharmacological pathways through which AOFs mitigate age-related decline are currently lacking.

The emergence of model organisms has revolutionized anti-aging research, with *Caenorhabditis elegans* (*C. elegans*), *Drosophila melanogaster*, *Danio rerio*, and *Mus musculus* serving as key biological models [30]. Among these, *C. elegans* offers unique experimental advantages: it is free from ethical constraints, possesses a short lifespan and well-characterized genome, and shares highly conserved gene signaling pathways with humans particularly those implicated in neurodegenerative and immune-related disorders [31]. Its transparent body, simple maintenance, and reproducible life cycle make *C. elegans* an excellent in vivo platform for anti-aging research and drug screening [9,14]. Complementing these biological models, network pharmacology has emerged as an innovative interdisciplinary approach integrating systems biology, computational biology, and network biology [32]. The selection and breeding of 82 *C. elegans* genes, which were identified through a systematic RNAi longevity screening, were based on an orthogonal study of human genes differentially expressed with age. This approach verified that *C. elegans* serves as a valuable comparative model for prioritizing human candidate aging genes [33]. Consequently, we employed network pharmacology to screen human targets and predict potential mechanisms of action. This approach enables the systematic elucidation of the complex interactions among active ingredients, molecular targets, and biological pathways, thereby providing new insights into the mechanisms of traditional Chinese medicine and facilitating modern drug discovery.

This study explores the anti-aging activity and underlying mechanisms of *Alpinia oxyphylla* polysaccharides (AOFs). Using *C. elegans* as a model organism, we systematically evaluated their anti-aging and antioxidant effects. Network pharmacology and molecular docking were further applied to identify key targets and pathways involved. Integrating experimental validation with computational analysis, this work reveals new mechanistic insights into the anti-aging potential of AOFs and highlights their promise as natural, sustainable candidates for next-generation green anti-aging therapeutics.

## 2. Results

### 2.1. Extraction and Characterization of AOFs

#### 2.1.1. Extraction, Separation, and Purification of AOFs

**Figure 1:**
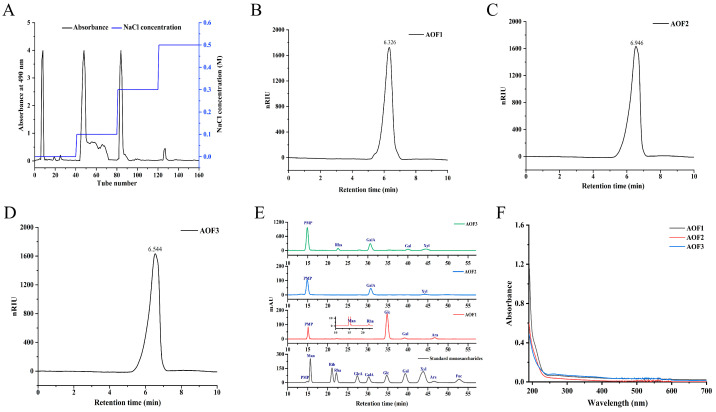
Extraction and characterization of *Alpinia oxyphylla* polysaccharides (AOFs). Elution curves of AOFs on DEAE-52 (**A**). High-performance gel permeation chromatography (HPGPC) atlas of AOF1 (**B**). HPGPC atlas of AOF2 (**C**). HPGPC atlas of AOF3 (**D**). Composition of monosaccharides of AOF1, AOF2, and AOF3 (**E**). Ultraviolet spectrum (**F**).

AOFs were obtained using the traditional water extraction and alcohol precipitation method, yielding 2.33% of crude polysaccharides. The crude AOFs were subsequently subjected to fractionation on a DEAE-52 cellulose ion-exchange column using stepwise elution with pure water and NaCl solutions at 0.1, 0.3, and 0.5 M, as illustrated in Figure 1A. Following elution, the four collected fractions were dialyzed and desalted, yielding four polysaccharide components, designated AOF1, AOF2, AOF3, and AOF4. Due to the limited quantity of AOF4, only AOF1, AOF2, and AOF3 were selected for subsequent chemical characterization, antioxidant assays, and anti-aging evaluations. The extraction rates of AOF1–3 polysaccharides for crude polysaccharides were 6.25%, 16.79% and 17.57%, respectively, and the extraction rates for samples were 0.61%, 1.64% and 1.71%, respectively. The primers are listed in Table S1.

#### 2.1.2. Analysis of the Monosaccharide Composition of AOFs

The molecular weights (Mw) of AOF1, AOF2, and AOF3 were determined by high-performance gel permeation chromatography (HPGPC). In HPGPC analysis, the retention time of a sample is inversely proportional to its molecular weight, with larger molecules eluting earlier. A dextran series (5, 12, 50, 150, 410, and 670 kDa) was used to generate a standard curve on the Shodex SUGAR KS-805 column, with retention time as the abscissa and logMw as the ordinate. The resulting linear regression equation was y = −7927x + 12.573 (R^2^ = 0.9933), which was used to calculate the molecular weights of the three polysaccharides based on their retention times (Figure 1B–D and Figure S1). As shown in Table S3, the calculated Mw values were 36,172 kDa, 11,665 kDa, and 24,298 kDa for AOF1, AOF2, and AOF3, respectively, indicating substantial differences in polymer size among the fractions.

Monosaccharide composition analysis revealed that AOF1 consisted predominantly of glucose (89.31%), along with mannose (0.46%), rhamnose (1.04%), galactose (3.91%), and arabinose (5.28%). As shown in Figure 1E, AOF2 was mainly composed of galacturonic acid (84.96%) and xylose (15.04%), whereas AOF3 contained arabinose (10.88%), galacturonic acid (54.70%), galactose (11.61%), and xylose (22.81%). Notably, glucose was the major monosaccharide in AOF1, while galacturonic acid predominated in AOF2 and AOF3, distinguishing these fractions from previously reported AOFs (AOFP) [28] and AO-derived polysaccharides (AOP) [29] obtained via alternative extraction methods, highlighting the novelty of these polysaccharide compositions.

#### 2.1.3. UV Spectroscopy Analysis of AOFs

The UV absorption spectra of the three polysaccharides were analyzed to assess potential contamination by nucleic acids and proteins, which exhibit characteristic absorption at 260 nm and 280 nm, respectively. As shown in Figure 1F, all three polysaccharides displayed only weak absorbance at these wavelengths, indicating that they lack detectable amounts of nucleic acids and proteins, or contain only trace quantities [34]. Importantly, these minimal levels do not interfere with subsequent chemical, antioxidant, or biological analyses.

### 2.2. Antioxidant Activity of AOFs In Vitro

#### 2.2.1. Analysis of DPPH Free Radical Scavenging Activity

**Figure 2:**
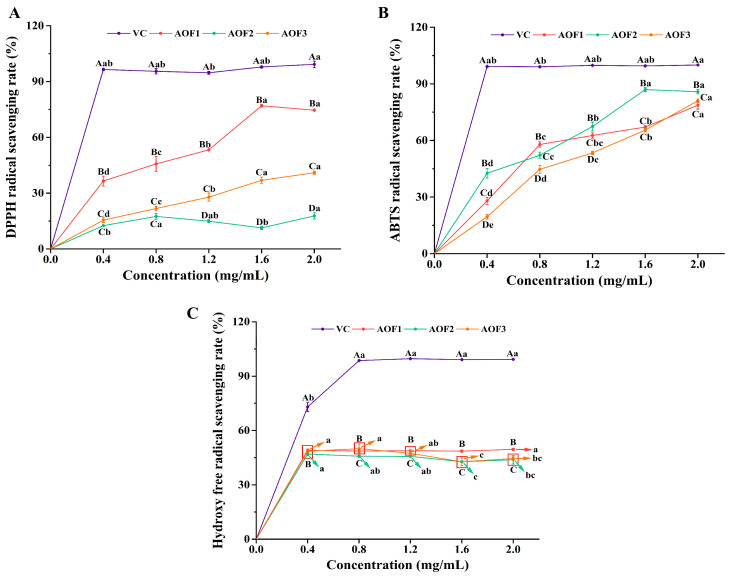
In vitro antioxidant activity of *Alpinia oxyphylla* polysaccharides (AOFs). DPPH free radical scavenging rate (**A**). ABTS free radical scavenging rate (**B**). Hydroxyl free radical scavenging rate (**C**). Different lowercase letters (a–e) denote significant differences across concentrations within the same group, whereas different uppercase letters (A–D) represent significant differences among various groups at the same concentration.

DPPH is a stable nitrogen-centered radical, and its unpaired electrons are neutralized upon reaction with antioxidants such as AOFs [35]. As shown in Figure 2A, the DPPH radical scavenging activity of AOF2 was relatively low and exhibited no clear concentration dependence, whereas AOF1 and AOF3 demonstrated significant concentration-dependent increases in scavenging activity, reaching maximal values at higher doses. Notably, the DPPH scavenging rate of AOF1 achieved 76.98% at the highest concentration, only 21.33% lower than that of vitamin C at the same dose. Previous studies have indicated that rhamnose (Rha) and glucose (Glu) play pivotal roles in maintaining antioxidant and anti-aging activities [36], both of which are uniquely abundant in AOF1, consistent with the observed results. Additionally, arabinose (Ara) has been reported to contribute to antioxidant and anti-aging effects [37], and acts as a potential regulator of lipid metabolism, a key factor in aging [38], providing a plausible explanation for the elevated DPPH radical scavenging activity observed in AOF3.

#### 2.2.2. ABTS Free Radical Scavenging Activity Analysis

The ABTS radical scavenging assay is widely employed to evaluate the electron- or hydrogen-donating capacity of antioxidants and is commonly used to assess the antioxidant activity of natural products via ABTS^+^ discoloration [39]. As shown in Figure 2B, all three AOF components exhibited significant concentration-dependent increases in ABTS radical scavenging activity, in contrast to the relatively concentration-independent behavior observed in the DPPH assay. At the highest concentrations, the scavenging activity of the polysaccharides approached that of vitamin C, reaching 86.96%. Notably, AOF2 displayed the strongest activity, which may be attributed to its exceptionally high galacturonic acid content. This observation aligns with the findings of Ke, Xin et al. [40], who reported that components with lower molecular weights and higher acidic sugar content exhibit enhanced ABTS radical scavenging activity, consistent with our monosaccharide analysis showing AOF2 contains 84.96% galacturonic acid.

#### 2.2.3. Analysis of Hydroxyl Radical Scavenging Activity

Hydroxyl radicals, one of the most reactive and harmful reactive oxygen species, interact capably with diverse biomolecules in living cells, ultimately causing cell damage and tissue injury [41]. As shown in Figure 2C, the hydroxyl radical scavenging activity of all three AOF components peaked at low concentrations (0.4 mg/mL). With increasing concentrations, the scavenging activity of AOF1 remained relatively stable, whereas that of AOF2 and AOF3 exhibited a slight decline (*p* < 0.05) to 42.75%, suggesting that even low concentrations of AOFs are sufficient to achieve substantial hydroxyl radical neutralization. This phenomenon may reflect a saturation effect at low concentrations, while at higher concentrations, the elevated content of acidic polysaccharides in AOF2 and AOF3 likely promotes intermolecular interactions, resulting in a modest reduction in scavenging efficiency.

In summary, variations in polysaccharide composition and structure contributed to differences in free radical scavenging among AOF1, AOF2, and AOF3. Nonetheless, all three polysaccharide components demonstrated appreciable antioxidant activity, corroborating the subsequent antioxidant and anti-aging assays and providing a theoretical foundation for further investigation.

### 2.3. Evaluation of the Anti-Aging Activity of AOFs on C. elegans

#### 2.3.1. Effects of AOFs on *C. elegans* Lifespan

**Figure 3:**
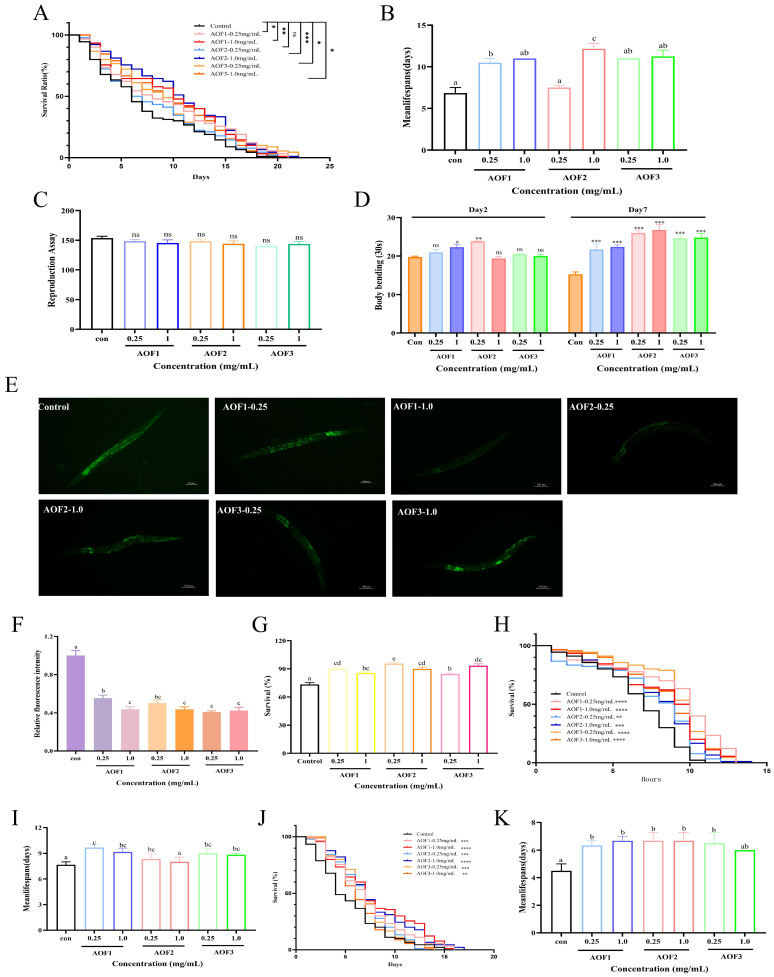
Effect of *Alpinia oxyphylla* polysaccharides on the lifespan of *Caenorhabditis elegans* (*C. elegans*). Determination of survival rate curve (**A**). Calculation of median lifespan (**B**). Reproduction assay (**C**). Measurement of body bending (**D**). Representative fluorescence photography of *C. elegans* (**E**). Relative fluorescence intensity of *C. elegans* lipofuscin (**F**). Determination of oxidative stress (**G**). Survival rate curve under heat stress (**H**). Median lifespan under heat stress conditions (**I**). Survival rate curve under ultraviolet irradiation (**J**). Median lifespan expectancy under ultraviolet irradiation (**K**). Different lowercase letters (a–e) denote significant differences. Where * represents *p* < 0.05, ** represents *p* < 0.01, *** represents *p* < 0.001, **** represents *p* < 0.0001 and ns represents *p* > 0.05.

Aging is a complex biological process characterized by a gradual decline and dysregulation of physiological functions, ultimately leading to death [42]. Lifespan serves as a direct and widely accepted biological indicator for assessing the aging process and evaluating interventions that improve physiological health in *C. elegans*. In the present study, the anti-aging potential of AOF1, AOF2, and AOF3 polysaccharides was evaluated by examining their effects on lifespan extension. The results demonstrated that exposure to AOF1 at 0.25 and 1.0 mg/mL increased the average lifespan of *C. elegans* by 27.75% and 29.12%, respectively (Figure 3A,B). Similarly, AOF2 at the same concentrations enhanced the average lifespan by 8.98% and 39.45%, whereas AOF3 treatment resulted in increases of 16.12% and 26.67%, respectively. These findings indicate that all three AOF components significantly prolonged lifespan in a dose-dependent manner. Notably, the concentration-dependent effects observed for AOF1, AOF2, and AOF3 are consistent with previous reports highlighting the potent anti-aging activities of plant-derived polysaccharides [43], further supporting their potential as natural agents for delaying aging.

#### 2.3.2. Effects of AOFs on the Healthy Lifespan of *C. elegans*

The growing demand for anti-aging interventions extends beyond merely delaying aging; there is an urgent need for safe and effective strategies to maintain the health of the elderly and prolong healthy lifespan. In *C. elegans*, key health parameters such as lipofuscin accumulation, reproductive capacity, and locomotor activity serve as reliable indicators of physiological status. Lipofuscin, an oxidative byproduct of lysosomal degradation, impairs cellular metabolism and reduces organ function, and its accumulation is widely recognized as a biomarker of aging. Previous studies have demonstrated that lipofuscin gradually accumulates with age in both *C. elegans* and humans, leading to a progressive increase in fluorescence intensity [44]. In the present study, treatment with AOF1, AOF2, and AOF3 at various concentrations significantly reduced lipofuscin fluorescence intensity compared with the control, with reductions of 44.73%, 56.27%, 49.89%, 56.36%, 59.19%, and 61.09%, respectively (Figure 3E,F). This concentration-dependent decrease in lipofuscin aligns with prior findings, suggesting that improved physiological function is associated with lifespan extension [45]. Reproductive capacity, reflected by egg production, is an important indicator of fitness in *C. elegans*. Assessment of fertility revealed that AOF1, AOF2, and AOF3 at all tested concentrations did not adversely affect oviposition, indicating that lifespan extension by these polysaccharides does not compromise reproductive ability (Figure 3C). Furthermore, locomotor activity, an indicator of muscle integrity and overall quality of life [40], was enhanced in treated worms, as evidenced by increased head swing frequency on day 7 (Figure 3D), reflecting improved muscle function and motility. The composition of monosaccharides is an important factor in the activity of polysaccharides, and the proportions and types of different monosaccharides in polysaccharides have a significant impact on their biological activity. In the context of anti-aging research, CAP, a polysaccharide, has been shown to significantly extend the lifespan of worms under oxidative and heat stress conditions. This effect may be attributed to the ratio of mannose to glucose (approximately 3:2), as polysaccharides with higher mannose content exhibit greater free radical scavenging capacity [46]. Glucose, mannose, arabinose, and uronic acid may play a key role in determining the superior antioxidant and anti-aging activity of natural polysaccharides. Collectively, these results demonstrate that AOFs not only significantly extend lifespan but also enhance health span in *C. elegans*. By reducing lipofuscin accumulation, preserving reproductive capacity, and improving locomotor function, AOFs mitigate age-related physiological decline, highlighting their potential as natural agents for promoting healthy aging.

#### 2.3.3. Effects of AOFs on Stress Resistance of *C. elegans*

In *C. elegans*, a positive correlation between longevity and stress resistance has been established, suggesting that enhanced resilience to environmental stressors contributes to lifespan extension [47]. Given that resistance to acute stress is closely linked to longevity, the ability of organisms to withstand external stimuli is a critical determinant of anti-aging efficacy. Heat stress, oxidative stress, and ultraviolet (UV) irradiation are known to accelerate cellular oxidation, thereby promoting rapid aging [48,49]. Accordingly, interventions that improve stress tolerance are pivotal for delaying aging.

In the present study, treatment with AOF markedly enhanced the survival of *C. elegans* under heat stress. Specifically, exposure to AOF1, AOF2, and AOF3 at concentrations of 0.25 and 1.0 mg/mL increased the average lifespan by 32.84%, 20.46%, 21.62%, 7.74%, 11.55%, and 13.86%, respectively, relative to the control. Correspondingly, as shown in Figure 3H,I, the maximum lifespan was extended by 25.81%, 22.58%, 16.12%, 19.35%, 25.81%, and 22.58%, respectively. These treatments consistently shifted the survival curves to the right, indicating enhanced thermotolerance.

Figure 3J,K showed that under UV irradiation, administration of AOF1, AOF2, and AOF3 at varying concentrations increased the average lifespan by 18.75%, 25%, 25%, 25%, 25%, 21.88%, and 12.50% compared with controls, with survival curves similarly shifting to the right, demonstrating improved resilience against UV-induced stress. Furthermore, under paraquat-induced oxidative stress, all treatment groups exhibited significantly higher survival rates relative to controls, confirming the protective effects of AOF against oxidative damage (Figure 3G).

Collectively, these results indicate that AOF1, AOF2, and AOF3 polysaccharides enhance *C. elegans* resistance to heat stress, UV irradiation, and oxidative stress, thereby contributing to lifespan extension. Notably, AOF1 consistently demonstrated superior anti-aging effects at equivalent concentrations, highlighting its potential as the most effective component among the three tested polysaccharides.

#### 2.3.4. Effects of AOFs on the Antioxidant Capacity of *C. elegans*

**Figure 4:**
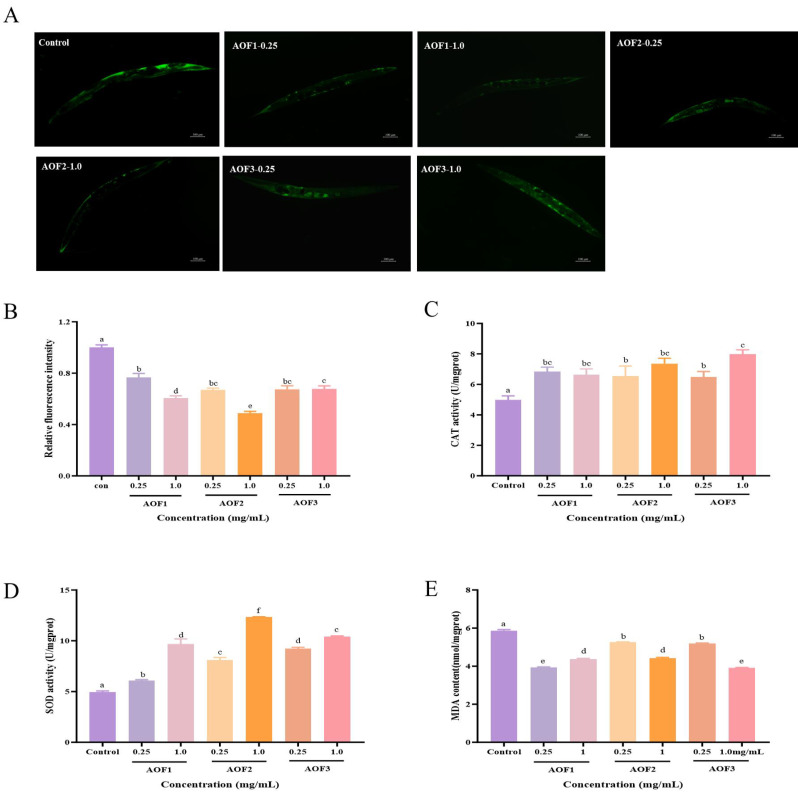
Representative fluorescence photographs of *Caenorhabditis elegans* (*C. elegans*) (**A**). Relative fluorescence intensity of reactive oxygen species (ROS) of *C. elegans* (**B**). Catalase (CAT) activity (**C**). Superoxide dismutase (SOD) activity (**D**). Malondialdehyde (MDA) activity (**E**). The different letters in each column represent significant differences between different groups (*p* < 0.05).

Aging is closely associated with the redox system in organisms, and *C. elegans* possesses a range of antioxidant defense enzymes, including superoxide dismutase (SOD), catalase (CAT), and peroxidases, which collectively mitigate oxidative damage induced by excessive reactive oxygen species (ROS) [50]. In the present study, treatment with AOF1, AOF2, and AOF3 significantly decreased ROS levels in *C. elegans*, as evidenced by reduced fluorescence intensity, with ROS reductions of 23.35%, 39.39%, 33.11%, 51.19%, 32.45%, and 32.22%, respectively (Figure 4A,B). Correspondingly, CAT activity increased by 37.20%, 23.26%, 31.16%, 47.91%, 30.23%, and 60% (Figure 4C), while SOD activity was enhanced by 22.19%, 94.87%, 62.99%, 148.35%, 85.87%, and 109.93%, respectively (Figure 4D). These results indicate that AOFs stimulate the antioxidant enzyme system in *C. elegans*, facilitating the clearance of superoxide radicals and strengthening resistance to oxidative stress.

Malondialdehyde (MDA) is a key biomarker of lipid peroxidation and reflects the degree of oxidative tissue damage. Treatment with AOF1, AOF2, and AOF3 resulted in significant reductions in MDA levels, with decreases of 32.6%, 25.30%, 10.49%, 24.69%, 11.71%, and 33.25%, respectively (Figure 4E), indicating a marked attenuation of lipid peroxidation. Collectively, these findings demonstrate that AOF1, AOF2, and AOF3 enhance oxidative stress resistance in *C. elegans* by reducing ROS accumulation, increasing antioxidant enzyme activity, and mitigating lipid peroxidation. The AOF component significantly increased the activities of SOD and CAT and the antioxidant enzymes associated with ROS scavenging, and reduced the content of MDA. By improving antioxidant enzyme activity, reducing lipid peroxidation, enhancing the body’s resistance to stress damage, and effectively prolonging the lifespan of nematodes. It is not difficult to see that in the above results, it can be found that low concentrations of AOF1 polysaccharides combine antioxidant capacity and anti-aging effects.

Notably, these results are consistent with prior findings on longan polysaccharides, where LP and LP-A significantly elevated SOD and CAT activities, reduced MDA content, and conferred anti-aging effects [40]. As the main component of AOF1, the antioxidant activity of glucose is consistent with previous research results [51]. Galacturonide can also confer excellent antioxidant capacity, while the galacturonic acid content of AOF3 is lower than that of AOF2 but has better antioxidant activity, which is presumed to be the synergistic antioxidant effect of arabinose and galactose.

The present study further suggests that AOF1, AOF2, and AOF3 effectively prolong the lifespan of *C. elegans* through enhancement of antioxidant defenses, reduction in lipid peroxidation, and improved stress tolerance. Of particular interest, low concentrations of AOF1 exhibited a synergistic effect, simultaneously promoting antioxidant capacity and anti-aging activity at equivalent doses.

#### 2.3.5. Effects of AOFs on the Expression of Anti-Aging Genes

**Figure 5:**
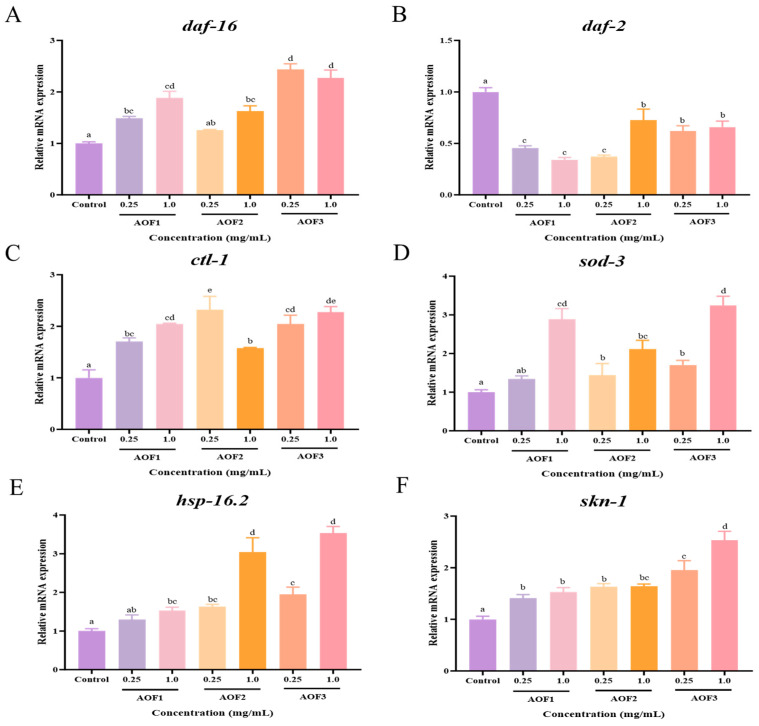
Effects of AOFs on the expression of anti-aging genes. Effect on daf-16 expression (**A**). Effects on daf-2 expression (**B**). Effect on ctl-1 expression (**C**). Effects on sod-3 expression (**D**). Effect on hsp-16.2 expression (**E**). Effect on skn-1 expression (**F**). Different lowercase letters (a–e) indicate significant differences among groups (*p* < 0.05).

The insulin/insulin-like growth factor signaling (IIS) pathway is a highly conserved regulatory pathway intimately associated with aging, observed in organisms ranging from *C. elegans* and *Drosophila* to humans. To investigate whether *Alpinia oxyphylla* polysaccharide components (AOF1, AOF2, and AOF3) delay aging via the IIS pathway, the expression of IIS-related genes in *C. elegans* was analyzed using RT-qPCR [52]. As shown in Figure 5A–F, treatment with different concentrations of AOF1, AOF2, and AOF3 led to a significant downregulation of *daf-2* mRNA levels, whereas the transcription of *daf-16*, *skn-1*, *sod-3*, *ctl-1*, and *hsp-16.2* genes was markedly upregulated.

The IIS pathway is pivotal in regulating growth, metabolism, development, longevity, and stress resistance in *C. elegans*. *DAF-16/FoxO*, a key downstream transcription factor of the IIS pathway, modulates the expression of genes involved in stress resistance and lifespan regulation. Activation of *DAF-16* enhances the transcription of antioxidant genes, including *SOD-3* and *CTL-1*, thereby stimulating antioxidant enzyme activity, promoting ROS clearance, and extending lifespan while improving stress resilience. Consistently, AOF1, AOF2, and AOF3 increased antioxidant enzyme activity and reduced ROS levels by activating *DAF-16*.

Furthermore, *SKN-1* and *DAF-16* are co-regulated by common upstream kinases and jointly modulate the transcription of phase II detoxification enzymes, enhancing oxidative stress resistance [53]. In the present study, AOF treatment upregulated *skn-1* expression, further improving *C. elegans* tolerance to oxidative stress. Heat stress responses, characterized by the induction of heat shock proteins, protect cells from deformation and enhance thermotolerance [54]. AOF1, AOF2, and AOF3 significantly increased *hsp-16.2* expression, thereby augmenting heat stress resistance in *C. elegans*.

In summary, AOF1, AOF2, and AOF3 effectively extend the lifespan of *C. elegans* through activation of the IIS pathway, enhancing antioxidant enzyme activities, improving stress resistance, reducing ROS accumulation and lipofuscin levels, decreasing lipid peroxidation, and alleviating oxidative damage in vivo.

#### 2.3.6. Bioinformatics Prediction and Analysis of DAF-16/SKN-1 Binding Sites

Based on sequence logo analysis, this study systematically constructed the DNA binding motifs of two core longevity transcription factors, DAF-16 and SKN-1, in *C. elegans* (Figure S2A,C). The core consensus sequence of the DAF-16 motif is TGTTTA, in which the relative information content (characterized by ΔΔG/RT values) of the guanine (G) at position 3 and the thymines (T) at positions 4–6 is significantly enriched. This sequence is highly consistent with previously reported recognition sequences for DAF-16/FOXO family transcription factors [55]. The core consensus sequence of the SKN-1 motif is TATCA, with the thymine residues at positions 2 and 4 and the cytosine (C) at position 5 exhibiting a high degree of sequence conservation. This is consistent with the role of SKN-1, as a homolog of Nrf2, in regulating downstream target genes through the antioxidant response element (ARE) [56]. Together with the DAF-16 motif, these sequences constitute the structural basis for the specific recognition of downstream target gene promoter regions by DAF-16 and SKN-1, respectively.

To further dissect the downstream regulatory networks of DAF-16 and SKN-1 in the insulin/IGF-1 signaling (IIS) pathway and the oxidative stress response pathway, this study performed a full-sequence scan and binding site prediction on the promoter regions of differentially up-regulated genes identified by qPCR using the above-mentioned motifs. The results showed that the 5′ upstream regulatory regions of the key antioxidant gene SOD-3 (superoxide dismutase-3), the heat shock protein-encoding gene HSP-16.2, and the catalase gene CTL-1 all contain potential binding sites for both DAF-16 and SKN-1. This suggests that these two transcription factors may co-regulate the above stress-responsive genes through either synergistic or independent mechanisms, which is consistent with previous findings that DAF-16 and SKN-1 form a regulatory crosstalk under oxidative stress conditions [57]. Specifically, two high-confidence DAF-16 binding sites were identified in the *SOD-3* promoter: a proximal site at −60 to −51 bp with the sequence TTTGTTTTA (score 11.741) and a distal site at −1855 to −1846 bp with the sequence ATTGTTTTA (score 11.501). In the *HSP-16.2* promoter, two binding sites were also found in the distal region at −1929 to −1920 bp (sequence AATGTTTTT, score 10.166) and at −806 to −797 bp (sequence AGTGTTATG, score 10.163). Additionally, one DAF-16 binding site was specifically identified in the distal region of the CTL-1 promoter at −1176 to −1167 bp, with the sequence TGTGTTTTA and a score of 10.571 (Figure S2B). These results are consistent with the molecular mechanism reported by Murphy et al., in which DAF-16 directly regulates antioxidant genes such as *sod-3*, *hsp-16.2*, and *ctl-1* [58]. Meanwhile, two SKN-1 binding sites were predicted in the proximal region (−172 to −132 bp) of the SOD-3 promoter, with the sequences ATTATCTTCAATAT and ATTCTCACGCTTTT and scores of 11.373 and 13.705, respectively. Two additional binding sites were also present in its distal region (near −759 bp and −694 bp), with the sequences TTTGTCTTCTTTGG and TAAATCAGCATCTT and scores of 9.105 and 12.129, respectively. In the distal region of the HSP-16.2 promoter, two SKN-1 binding sites were identified at coordinates −1433 to −1420 bp and −973 to −960 bp, with the sequences GACGTCATCATTTT and GACGTCATCATCTC and scores of 14.364 and 10.882, respectively. Furthermore, two potential SKN-1 binding sites were also found in the distal region of the CTL-1 promoter (at −1433 to −1420 bp and −973 to −960 bp), with scores of 14.364 and 10.882, respectively (Figure S2D). The recognition of these antioxidant gene promoters by SKN-1 is similar to its mechanism of activating the expression of phase II detoxification genes such as gcs-1 and gst-4 via the ARE under oxidative stress conditions [59]. The list of potential transcription factor binding sites in the promoter region of the specific target gene is shown in Table S4, including site sequence, location, strand orientation and matching score.

In summary, DAF-16 and SKN-1 may bind to distinct cis-acting elements within the promoter regions of SOD-3, HSP-16.2, and CTL-1, thereby establishing crosstalk in the transcriptional regulatory network and synergistically activating the transcription of genes involved in antioxidant defense and proteostasis. This, in turn, integrates downstream signals from the IIS pathway and the oxidative stress response pathway [60]. Notably, the CTL-1 promoter contains binding sites for both DAF-16 and SKN-1, suggesting that this gene may serve as a key node co-regulated by these two transcription factors, playing a central role in catalase-dependent reactive oxygen species (ROS) clearance and redox homeostasis maintenance. This observation is consistent with previous reports on the regulation of DAF-16 and SKN-1 pathways in alleviating oxidative stress [57]. Together, these results provide important target gene validation evidence for further elucidating the molecular mechanism by which DAF-16 and SKN-1 cooperatively regulate anti-aging processes in *C. elegans*.

### 2.4. Network Pharmacology and Molecular Docking to Explore the Anti-Aging Molecular Mechanism of AOFs

#### 2.4.1. Target Prediction of AOFs to Improve Aging

Using multiple bioinformatics platforms, potential molecular targets of AOF1, AOF2, and AOF3 were systematically identified to elucidate their anti-aging mechanisms. Specifically, the SwissTargetPrediction database (http://swisstargetprediction.ch/, accessed on 20 June 2025), GeneCards (https://www.genecards.org/, accessed on 20 June 2025), and PharmMapper (https://www.lilab-ecust.cn/pharmmapper/index.html, accessed on 20 June 2025) were employed to predict the putative targets of the three polysaccharide fractions. As a result, 1065, 494, and 657 targets were obtained for AOF1, AOF2, and AOF3, respectively.

**Figure 6:**
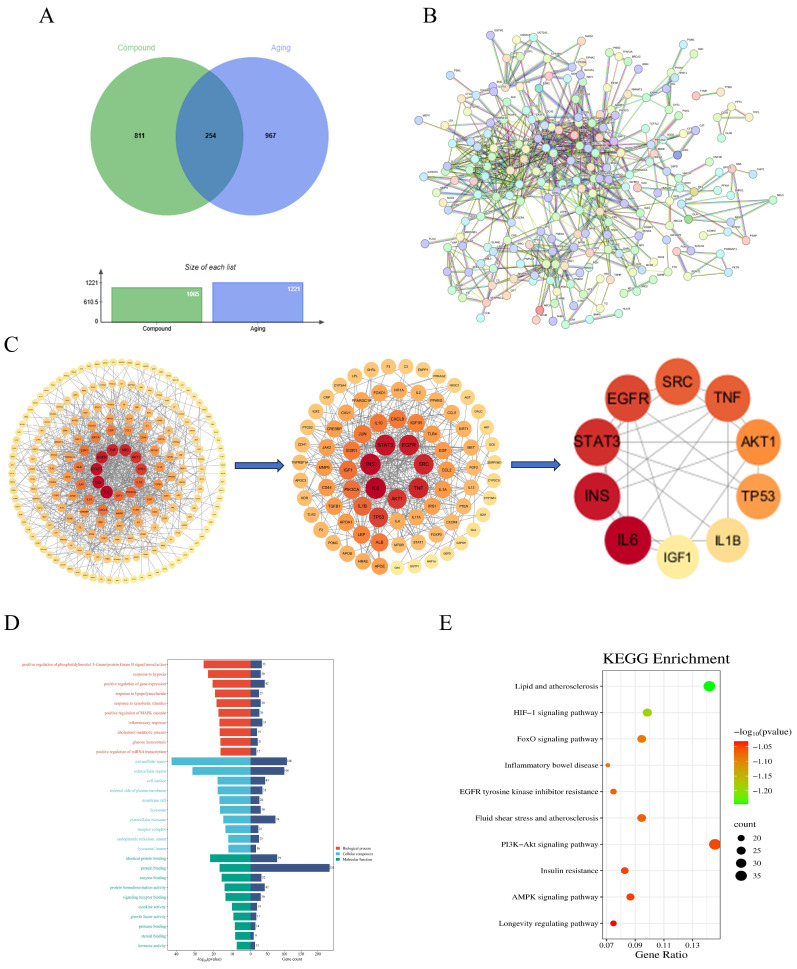
Network pharmacological analysis of AOF1. Wayne diagram (**A**). Protein–protein interaction (PPI) network diagram (**B**). Core target screening process (**C**). Analysis of biological processes, cellular components, and molecular functions (**D**). KEGG pathway analysis (**E**).

**Figure 7:**
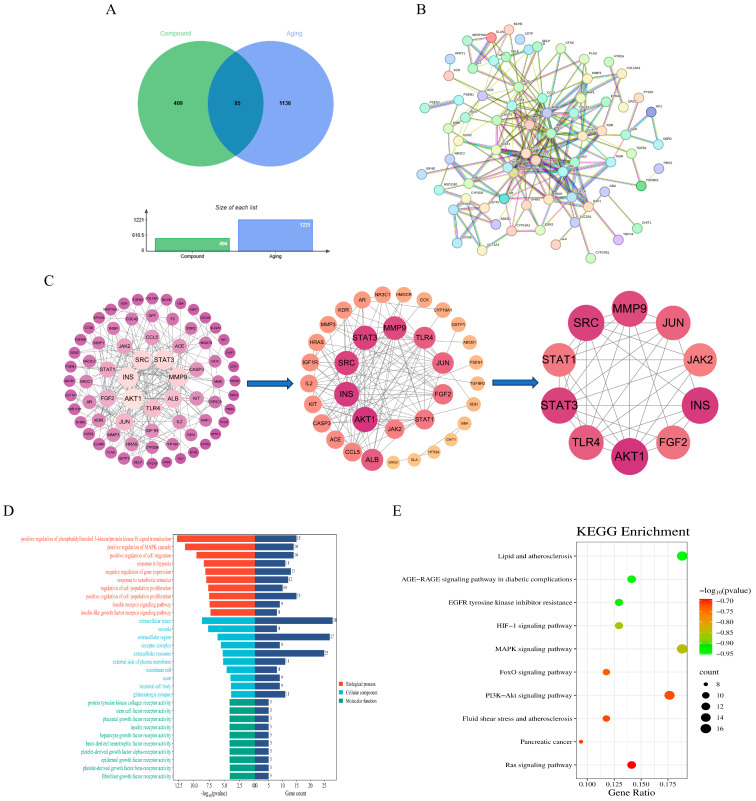
Network pharmacological analysis of AOF2. Wayne diagram (**A**). Protein–protein interaction (PPI) network diagram (**B**). Core target screening process (**C**). Analysis of biological processes, cellular components, and molecular functions (**D**). KEGG pathway analysis (**E**).

**Figure 8:**
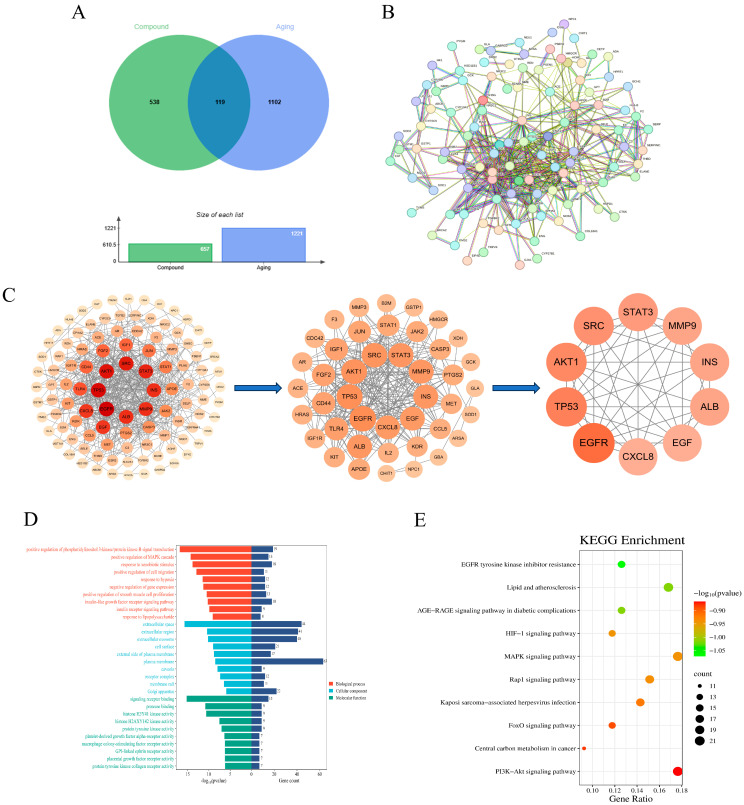
Network pharmacological analysis of AOF3. Wayne diagram (**A**). Protein–protein interaction (PPI) network diagram (**B**). Core target screening process (**C**). Analysis of biological processes, cellular components, and molecular functions (**D**). KEGG pathway analysis (**E**).

Concurrently, aging-related genes were retrieved from the GeneCards (https://www.genecards.org/, accessed on 20 June 2025), OMIM (https://www.omim.org/, accessed on 20 June 2025), and DrugBank Online (https://go.drugbank.com/, accessed on 20 June 2025) databases, yielding a total of 1221 aging-associated targets. To identify the common molecular mechanisms through which AOF1, AOF2, and AOF3 may exert anti-aging effects, the predicted compound targets were intersected with aging-related targets using Venny 2.1.0 (https://bioinfogp.cnb.csic.es/tools/venny/, accessed on 20 June 2025). The intersection analysis as visualized in Figure 6A, Figure 7A and Figure 8A, revealed 254, 85, and 119 shared targets for AOF1, AOF2, and AOF3, respectively.

These intersecting targets represent potential core genes mediating the anti-aging effects of AOF, providing a theoretical basis for subsequent network pharmacology and molecular mechanism analyses.

#### 2.4.2. PPI Network Analysis of Polysaccharide Intersection Targets of AOFs

To further elucidate the potential protein–protein interactions (PPI) among the intersecting targets of AOF1, AOF2, and AOF3, network analyses were conducted using the STRING database. The datasets containing 254, 85, and 119 intersecting gene targets corresponding to AOF1, AOF2, and AOF3, respectively, were uploaded to the platform to construct PPI networks. As illustrated in Figure 6B, the AOF1-related network comprised 254 nodes and 730 edges, with an average node degree of 5.75, indicating moderate connectivity among its targets. Similarly, the AOF2 network (Figure 7B) contained 85 nodes and 229 edges, with an average node degree of 5.39, while the AOF3 network (Figure 8B) was composed of 119 nodes and 492 edges, yielding an average node degree of 8.27, suggesting a comparatively denser interaction profile.

In these networks, larger and darker nodes represent proteins with stronger interaction intensity and higher connectivity, implying that such proteins may play pivotal regulatory roles within the anti-aging signaling framework of AOF. These interaction datasets were subsequently imported into Cytoscape10.3 software for advanced topological analysis. Based on the degree centrality parameter, the top ten key targets were identified for each polysaccharide fraction (Figure 6C, Figure 7C and Figure 8C). Comparative analysis revealed that INS, STAT3, SRC, and AKT1 shared hub targets across all three polysaccharide components.

These common key targets are hypothesized to mediate the core anti-aging mechanisms of AOF1, AOF2, and AOF3, highlighting their potential involvement in shared regulatory pathways and offering a foundation for subsequent molecular docking and pathway enrichment analyses.

#### 2.4.3. KEGG Enrichment Analysis of AOF Polysaccharide Gene Ontology (GO)

For comprehensive bioinformatics analysis, the identified core targets of AOF1 (254 genes), AOF2 (85 genes), and AOF3 (119 genes) were subjected to Gene Ontology (GO) and Kyoto Encyclopedia of Genes and Genomes (KEGG) pathway enrichment analyses to elucidate their potential biological functions and signaling pathways. As presented in Figure 6D, Figure 7D and Figure 8D, the GO enrichment results revealed that, at a threshold of *p*-adjust < 0.001, there were 259, 11, and 35 entries associated with biological processes (BPs), while at *p*-adjust < 0.05, there were 75, 34, and 55 entries for cellular components (CCs), and 126, 72, and 83 entries for molecular functions (MFs) in AOF1, AOF2, and AOF3, respectively. The top 10 enriched terms in each GO category were analyzed in detail.

In the BP category, enrichment terms closely associated with the aging process were identified across all three AOF, including positive regulation of phosphatidylinositol 3-kinase/protein kinase B (PI3K/AKT) signaling, positive regulation of MAPK cascade, response to hypoxia, and response to xenobiotic stimulus. These pathways are widely recognized as central regulators of aging, as dysregulation of PI3K/AKT and MAPK signaling has been shown to contribute to stem cell depletion, impaired angiogenesis, and loss of tissue homeostasis [61]. Moreover, the response to exogenous toxicants represents a critical intersection between environmental stress and accelerated organismal aging [62]. In the CC category, AOF1, AOF2, and AOF3 shared several key enrichment terms, such as extracellular space, extracellular region, receptor complex, and external side of plasma membrane, which are integral to cellular communication, metabolic regulation, and structural integrity. These components are often implicated in metabolic dysfunction, structural degeneration, and cellular senescence [63,64]. Additionally, enrichment of membrane raft and extracellular exosome terms suggests potential involvement in neurological aging processes and intercellular signaling, meriting further mechanistic exploration [65,66,67]. Within the MF category, although AOF1, AOF2, and AOF3 exhibited distinct profiles, several enrichment terms such as protein tyrosine kinase collagen receptor activity, epidermal growth factor receptor (EGFR) activity, protease binding, and insulin receptor activity were consistently associated with molecular mechanisms of aging.

To further clarify these biological implications, KEGG pathway enrichment analysis was performed (Figure 6E, Figure 7E and Figure 8E), identifying several statistically significant pathways common to all three polysaccharides, including lipid and atherosclerosis, EGFR tyrosine kinase inhibitor resistance, HIF-1 signaling pathway, FoxO signaling pathway, and PI3K-Akt signaling pathway. Collectively, these findings demonstrate that AOF1, AOF2, and AOF3 exert anti-aging effects through multi-target and multi-pathway mechanisms, suggesting a complex regulatory network that integrates oxidative stress resistance, metabolic regulation, and cellular signaling modulation.

### 2.5. Analysis of Homology Modeling and Molecular Docking

AOFs have been demonstrated to possess potent anti-aging activity; however, the underlying molecular mechanisms remain to be elucidated. To explore these mechanisms, molecular docking was performed to investigate the interactions between AOFs and key human anti-aging target proteins. In this study, the four crucial proteins ATK1, INS, SRC, and STAT3 were subjected to homology modeling and structural validation via Ramachandran plot analysis. As illustrated in Figure S3, more than 99.5% of the amino acid residues for each target protein were located within the favored regions of the Ramachandran plot, confirming the high reliability and structural accuracy of the models.

**Figure 9:**
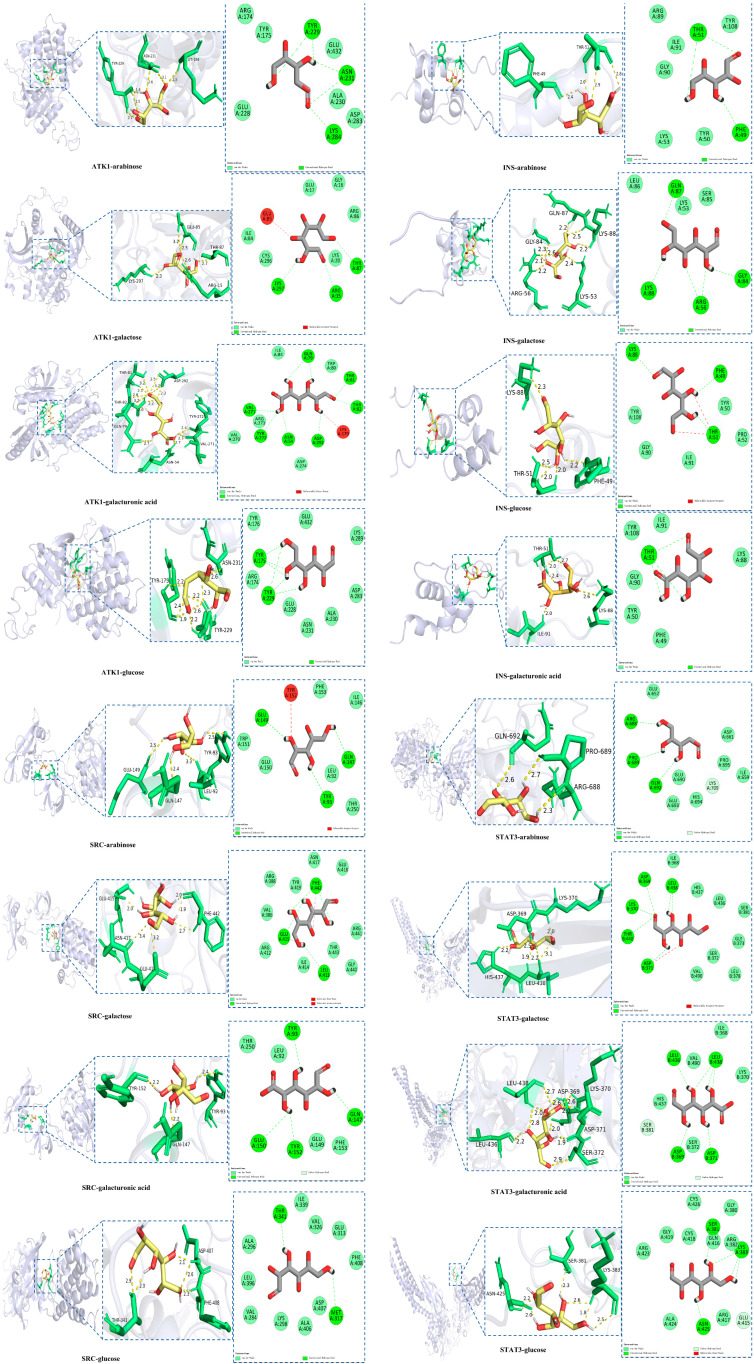
Molecular docking of the anti-aging key target proteins (ATK1, INS, SRC, and STAT3) and the critical monosaccharides (arabinose, galactose, galacturonic acid and glucose). Amino acid residue fragments are depicted by sticks, and hydrogen bonds are shown as yellow dotted lines in three-dimensional structures.

As shown in Table S5 and Figure 9 and Figure S5, the primary stabilizing forces within the receptor–ligand complexes were provided by hydrogen bonds, including both conventional and carbon–hydrogen bonds, as well as van der Waals forces. Hydrogen bonding, being the most prevalent electrostatic interaction, serves as the major stabilizing force within such receptor–ligand systems [68]. Although some complexes exhibited minor unfavorable receptor–ligand interactions, their overall binding energies remained below −3.9 kcal/mol, underscoring the dominant stabilizing role of hydrogen bonding and van der Waals interactions in the docking process. These findings are in line with the observations reported by [69]. Notably, several complexes demonstrated binding energies as low as −5.5 kcal/mol, indicating strong receptor–ligand affinity. Among all four proteins, INS exhibited comparatively lower binding energies, which may be attributed to its relatively simple receptor structure and fewer binding residues; nevertheless, these complexes formed spontaneously and maintained structural stability.

Figure 9 and Figure S5 illustrate the 3D and 2D docking conformations of four monosaccharides and metformin with the four target proteins, depicting their interaction patterns and binding sites. For ATK1, the docking results showed that sugars mainly bound through hydroxyl-mediated interactions, with occasional involvement of carbon–oxygen double bonds. Similar to the results of [70], the residues Tyr, Asn, Asp, and Thr played crucial roles in the recombination process, participating repeatedly across various monosaccharide complexes and forming multiple intermolecular interactions at distinct sites on each sugar molecule. Specifically, Tyr175, Tyr229, and Tyr272 were consistently engaged in hydrogen bonding with arabinose and glucose, indicating that tyrosine residues are key contributors to the formation of the ATK1 complex. Furthermore, Asn231 emerged as a critical site for interaction with arabinose, mannose, and xylose, engaging at least two functional groups of these ligands.

Although the absolute binding energy of the INS complex was relatively low, several key residues including Thr51, Phe49, and Lys88 played vital roles in stabilizing the interactions across multiple monosaccharide complexes. In particular, Thr51 contributed to polyhydrogen bond formation in three complexes. Despite minor unfavorable receptor–receptor interactions observed in the INS–glucose complex, the overall receptor–ligand binding remained spontaneous and stable. Similarly, for the SRC and STAT3 complexes, multiple stabilizing interactions were identified involving the residues Gln147 and Phe442. In addition, the residues Glu, Tyr and Thr were recognized as key residues in SRC complex formation, whereas Asp369, Asp371, Leu438 and Lys383 played essential roles in stabilizing the STAT3 complexes, providing multiple hydrogen bonding forces. Additionally, in the STAT3-mannose complex, two strong carbon–hydrogen bonds were identified, contributing significantly to the stability of the complex. It is also worth noting that the strong binding affinity of the STAT3 complex during docking may be related to some short hydrogen bonds. Additionally, in addition to the complexes formed by INS protein docking, the complexes formed by galactose and galacturonic acid with proteins are generally lower than that of the positive control metformin, which confirms that both of them play an important role in the biological process of anti-aging.

The hydrogen bond interaction and van der Waals force still play an important role in the formation of metformin complexes. For example, during the formation of INS metformin, two hydrogen bonds were formed at the residues of Phe49 and Thr51, which promoted the stable formation of the complex, which was also an important reason for its relatively high docking fraction. At the same time, the strong hydrogen bond at Gly409 with a length of 1.9 Å and the double hydrogen bond at Ala411 jointly promote the binding affinity of the SRC complex, while Ile711, as the key binding site in the docking process of the STAT3 complex, dominates the generation of a large number of hydrogen bonds.

In summary, all monosaccharide constituents and metformin exhibited stable and spontaneous binding to the four key anti-aging target proteins through extensive hydrogen bonding and van der Waals interactions. In addition, galactose and galacturonic acid may have more critical potential anti-aging activities than the positive control metformin. These molecular docking results provide a strong mechanistic foundation for the observed anti-aging activity of AOF and offer a detailed molecular explanation consistent with previous structural and biochemical studies.

### 2.6. Correlation Analysis

Aging and lifespan are closely associated with oxidative stress [71]. To further elucidate the relationships and underlying mechanisms among anti-aging processes, oxidative stress, and gene expression, a correlation analysis was performed (Figure S4), where red blocks represent positive correlations and blue blocks indicate negative correlations, with asterisks denoting levels of statistical significance (* *p* < 0.05, ** *p* < 0.01, *** *p* < 0.001). As expected, the accumulation of lipofuscin, a well-established biomarker of aging, exhibited a negative association with the lifespan of *C. elegans*, consistent with previous observations [45]. Simultaneously, elevated levels of reactive oxygen species (ROS), indicative of intensified oxidative stress and molecular damage, were found to promote lipofuscin accumulation (*p* < 0.01) [72]. Moreover, locomotor activity, reflecting the structural and functional integrity of nematode muscle, is directly related to overall quality of life [40]. Accordingly, increased oxidative damage was strongly correlated with the deterioration of muscular architecture and the decline in motility in *C. elegans* (*p* < 0.01). Conversely, enhanced superoxide dismutase (SOD) activity was associated with reduced levels of free radicals and increased activity of other antioxidant enzymes [73], ultimately contributing to an extension of health span (*p* < 0.05). The synergistic actions of SOD and catalase (CAT) effectively decreased lipofuscin accumulation and ROS activity, thereby alleviating oxidative stress, enhancing radio resistance, and improving muscular integrity and motor performance.

The observed strong positive correlations between locomotor capacity, CAT and SOD levels, and resistance to oxidative and UV stress indicate that robust antioxidant defenses are essential for preserving neuromuscular function. Previous studies have demonstrated that a decrease in *daf-2* function enables fertile adult *C. elegans* to remain active for substantially longer periods and extends lifespan by more than twofold [74]. Consistent with these findings, *daf-2* downregulation in our study promoted enhanced antioxidant activity and improved resistance to UV radiation. Notably, upregulation of *ctl-1* and *hsp-16.2* genes has been shown to activate a cascade of antioxidant enzymes, thereby improving longevity and stress tolerance in *C. elegans* [53,54]. In agreement with our observations, *ctl-1* expression diminishes lipofuscin deposition and augments antioxidant activity, whereas *hsp-16.2* enhances CAT and SOD activities, collectively strengthening the oxidative stress response.

In summary, aging, antioxidant defense, and organismal vitality are intrinsically interconnected. Aging represents a complex biological process governed by multiple genes, molecular targets, and regulatory factors. The modulation of lipofuscin levels and the activities of key antioxidant enzymes play a pivotal role in anti-aging mechanisms, serving as direct determinants of health span and longevity. These findings reveal underlying regulatory relationships not apparent from raw data alone, thereby expanding the mechanistic understanding of anti-aging processes and providing a theoretical basis for future research.

## 3. Discussion

In this study, three homogeneous polysaccharide components, AOF1, AOF2, and AOF3, were successfully isolated and purified from AO. In vitro activity assessments demonstrated that all three components exhibited significant antioxidant activity; however, their potency varied across different antioxidant models, reflecting the intricate structure–function relationships underlying their bioactivity.

Among the three components, AOF2 exhibited the strongest activity in ABTS and hydroxyl radical scavenging assays. As established in prior research, molecular weight is a key determinant of polysaccharide antioxidant activity [75]. In general, polysaccharides with lower molecular weight generally demonstrate superior free radical scavenging capacity, attributable to reduced steritic hindrance, enhanced solubility, and greater accessibility of reactive hydroxyl groups [76]. Consistent with this structure–activity principle, the relatively low molecular weight of AOF2 is likely a primary factor contributing to its pronounced efficacy in these two assays. Its monosaccharide profile may further contribute synergistically to this activity advantage. While AOF1 and AOF3 showed lower potency, their clear dose-dependent responses indicate stable radical scavenging potential, which may approach that of AOF2 at higher concentrations. Notably, the activity hierarchy shifted in the DPPH radical scavenging assay, where AOF1 emerged as the most active component. The scavenging mechanism for DPPH radicals differs from that for ABTS and hydroxyl radicals, exhibiting higher structural selectivity for hydrogen donors [77]. AOF1 was characterized by a high content of rhamnose, glucose, and arabinose. The presence of rhamnose has been associated with enhanced radical scavenging [78], while glucose and arabinose are commonly identified as active monosaccharides contributing to antioxidant activity [79]. Consequently, we infer that the unique and potentially more complex monosaccharide composition, glycan chain structure, molecular weight, degree of branching, glycosidic linkage types, and conformation of AOF1 provide additional active sites for interaction with the DPPH radical, accounting for its superior performance in this specific model. This structural rationale also explains the comparable activity of AOF3 in the DPPH system, suggesting it may share certain key active structural units with AOF1.

The findings of this study align with the fundamental principle of the “structure–activity relationship of polysaccharides.” The differences in antioxidant activity among the distinct polysaccharide components result from the integrated effects of multiple structural factors, including molecular weight, monosaccharide composition, glycosidic bond type, degree of branching, and even higher-order spatial conformation [79,80]. The activity of AOF2 appears to be predominantly influenced by its molecular weight, whereas the activity of AOF1 may rely more heavily on its specific monosaccharide sequence and glycan chain configuration. This differentiated performance across models not only underscores the potential of AOFs as a source of natural antioxidants but also suggests that individual components may be uniquely suited to counteract specific types of oxidative stress and damage.

This study systematically evaluated the anti-aging activity and underlying mechanisms of the polysaccharide components AOF1, AOF2, and AOF3 from AOFs in the model organism *C. elegans*. The results demonstrated that all three polysaccharide components, at a low concentration (0.25 mg/mL), significantly extended the mean lifespan of *C. elegans* by 32.84%, 21.62%, and 11.55%, respectively. Moreover, they effectively improved survival under both thermal stress and oxidative stress conditions, suggesting their potential to enhance organismal stress resistance. Notably, current research on anti-aging is gradually shifting from a paradigm focused merely on “lifespan extension” toward one emphasizing the promotion of “healthy aging.” Traditional interventions often achieve lifespan prolongation at the cost of reduced reproductive capacity or compromised tissue integrity [40,81]. In contrast, the present study revealed that AOFs not only extended lifespan but also significantly reduced lipofuscin accumulation and maintained muscle architecture integrity in *C. elegans*. Importantly, no obvious adverse effects on physiological functions were observed. These findings indicate that AOFs can prolong health span without compromising health status, demonstrating a favorable biosafety profile.

At the mechanistic level, this study provides further support for the core tenet of Harman’s free radical theory of aging, which posits that oxidative stress is one of the key drivers of the aging process [82]. AOFs exert their antioxidant and anti-aging effects through multiple mechanisms. First, they act as direct free radical scavengers. In vitro assays demonstrated that AOF1, AOF2, and AOF3 exhibit significant scavenging capacities against ABTS, DPPH, and hydroxyl radicals, indicating their ability to directly reduce ROS levels via non-enzymatic pathways. Second, AOFs enhance the endogenous antioxidant system. Under oxidative stress conditions, all three polysaccharide components significantly increased the activities of SOD and CAT while reducing MDA content, thereby alleviating lipid peroxidation damage and improving the oxidative stress tolerance of *C. elegans*. Third, based on network pharmacology or molecular docking, we predict they likely modulate the insulin/IGF-1 signaling (IIS) pathway. Although direct activity measurements of the pathway were not performed in this study, based on previous findings, it is plausible that AOFs may downregulate IIS, thereby activating the FOXO/DAF-16-mediated antioxidant response and synergistically enhancing organismal stress resistance.

Finally, our findings demonstrate that AOF1, AOF2, and AOF3 suppress the expression of upstream genes *daf-2* and *age-1*, while upregulating the expression of genes including *daf-16*, *sod-3*, *skn-1*, *ctl-1*, and *hsp-16.2*. The IIS pathway plays a pivotal role in multiple biological processes in *C. elegans*, including growth, metabolism, development, lifespan, and stress resistance [83]. DAF-16/FOXO is a crucial transcription factor that regulates genes associated with stress resistance and longevity. Its translocation into the nucleus can be triggered by various stress stimuli, such as oxidative stress, which subsequently modulates the expression of stress-responsive genes. Activated DAF-16 enhances the expression of downstream antioxidant genes (e.g., *sod-3* and *ctl-1*), thereby activating a series of antioxidant enzymes, increasing their activity, and ultimately extending lifespan and bolstering stress resistance in worms [84]. By activating *daf-16*, AOF1, AOF2, and AOF3 elevate antioxidant enzyme activity and improve ROS clearance. Moreover, *skn-1* and *daf-16* are regulated by a shared kinase cascade; modulating the transcriptional levels of phase II metabolic enzymes through this axis can enhance the organism’s resistance to oxidative stress [53]. AOF1, AOF2, and AOF3 upregulate *skn-1* expression, thereby strengthening the oxidative stress resilience of *C. elegans*. Additionally, under thermal stress, cells mount a heat shock response, inducing the expression of genes such as *hsp-16.2* to prevent protein misfolding and enhance thermotolerance [85]. We observed that AOF1, AOF2, and AOF3 improve the heat stress resistance of *C. elegans*, and further PCR analysis revealed that this effect is mediated through the upregulation of the heat shock-related gene *hsp-16.2*. Prediction of transcription factor binding sites indicated that DAF-16 and SKN-1 may bind to distinct cis-acting elements within the promoter regions of SOD-3, HSP-16.2, and CTL-1, thereby establishing crosstalk in the transcriptional regulatory network and synergistically activating the transcription of genes involved in antioxidant defense and proteostasis. This provides important target gene validation evidence for further elucidating the molecular mechanism by which DAF-16 and SKN-1 cooperatively regulate anti-aging processes in *C. elegans*. In summary, AOF1, AOF2, and AOF3 promote DAF-16 nuclear translocation, increase antioxidant enzyme activity, enhance stress resistance, reduce intracellular ROS levels and lipofuscin accumulation, attenuate lipid peroxidation, and mitigate oxidative damage by modulating the IIS pathway, thereby effectively extending the lifespan of *C. elegans.* Although we observed upregulation of mRNA levels of several genes, including sod-3 and ctl-1, the current data do not directly demonstrate nuclear translocation of the DAF-16 protein. Confirming the actual translocation and transcriptional activity of DAF-16 represents a necessary next step in future studies.

This study preliminarily clarifies the differential in vitro antioxidant activities of various AOF polysaccharide fractions and their potential structural causes, yet limitations persist, including an incomplete structural characterization, currently limited to monosaccharide composition and molecular weight, and a lack of in vivo validation for the observed bioactivity. In the network pharmacology component, target prediction was conducted based on the monosaccharide composition of AOFs. This simplified analytical framework fails to account for the in vivo absorption and metabolic fate of polysaccharides as macromolecular polymers. Consequently, the results should be interpreted as exploratory rather than conclusive. Predictions derived from monosaccharide profiles cannot substitute for direct mechanistic investigations into the intact polysaccharide molecule. Accordingly, future studies will prioritize the use of full-length AOF, aiming to provide a more rigorous methodological foundation. Network pharmacology and molecular docking only provide initial and rapid predictions of related mechanisms, lacking verification. Subsequent experiments will add protein experiments, molecular dynamics, and other related methods to verify and enrich the mechanism explanations based on this foundation. Future research should prioritize elucidating the structure–activity relationship through precise structural analysis using techniques such as methylation analysis, NMR, and AFM, and systematically reveal the underlying molecular mechanisms by investigating relevant signaling pathways (including Nrf2/ARE, SIRT1). This will establish a theoretical foundation for the high-value development of AOFs and pave the way for designing targeted functional polysaccharide products with potential applications as natural antioxidants or anti-aging agents.

## 4. Materials and Methods

### 4.1. Experimental Materials and Reagents

The *C. elegans* wild-type strain N2 and its standard food source *Escherichia coli* OP50 were used in this study. Reactive oxygen species (ROS) detection kits, malondialdehyde (MDA; lot no. BC0025), superoxide dismutase (SOD; lot no. BC0175), and catalase (CAT; lot no. BC0205) assay kits were obtained from Beijing Solarbio Science & Technology Co., Ltd. (Beijing, China). Methyl violet (lot no. 119824BB) and 5-fluoro-2′-deoxyuridine (lot no. 61000A) were used as experimental reagents. Total RNA extraction reagent was purchased from Kunming Yungeng Biotechnology Co., Ltd. (Kunming, China). All other chemicals and solvents were of analytical or chromatographic grade and procured from local certified suppliers.

### 4.2. Extraction and Characterization of AOFs

#### 4.2.1. Extraction and Purification of AOFs

The *Alpinia oxyphylla* polysaccharides (AOFs) used in this study were sourced from the Kang’an Hall Traditional Chinese Medicine Center (Kunming, China). The extraction procedure was conducted with minor modifications based on a previously reported method [52]. Briefly, *A. oxyphylla* (AO) fruits were ground into fine powder, passed through a 60-mesh sieve, and defatted by triple extraction with 85% ethanol. The defatted material was subsequently extracted three times with distilled water at 70 °C using a solid-to-liquid ratio of 1:15 (*w*/*v*). The combined extracts were concentrated to the desired volume and precipitated overnight with four volumes of ethanol at room temperature. The resulting precipitate was collected by centrifugation (4000 rpm, 15 min, 0 °C), redissolved in hot water, and deproteinized using the Sevag method. The protein-free extracts were then freeze-dried to obtain the crude AO polysaccharides.

Calculate the extraction rate (extraction rate % = crude polysaccharide quality/tea raw material quality × 100).

For purification, the crude AOFs were fractionated on a DEAE-52 cellulose column and sequentially eluted with distilled water and NaCl solutions at concentrations of 0.1, 0.3, and 0.5 mol/L. The elution endpoints were determined using the phenol–sulfuric acid method. Each eluent was collected, concentrated, dialyzed, and lyophilized to yield four distinct AOF fractions, designated as AOF1, AOF2, AOF3, and AOF4.

Further purification was performed using a Sephadex G-100 column (1.9 × 110 mm) with distilled water as the eluent at a flow rate of 0.2 mL/min. Fractions (3.2 mL/tube) were collected using an automated fraction collector(Shanghai Qingpu Huxi Instrument Factory, Shanghai, China), and polysaccharide content was monitored by high-performance liquid chromatography coupled with a refractive index detector (HPLC-RID) (Agilent Technologies, Inc., Santa Clara, CA, USA). Fractions exhibiting identical elution profiles were pooled, concentrated, and dialyzed with a 1000 Da molecular weight cutoff membrane. The resulting homogeneous polysaccharide fractions (AOF1, AOF2, and AOF3) were obtained by lyophilization and stored at low temperature for subsequent analyses.

#### 4.2.2. Determination of Uniformity and Molecular Weight

The molecular weight distribution and homogeneity of the purified polysaccharide fractions (AOF1, AOF2, and AOF3) were determined using high-performance gel permeation chromatography (HPGPC). Analyses were performed on an Agilent 1260 HPLC system equipped with a refractive index detector (RID). Separation was achieved using a Shodex SUGAR KS-805 gel filtration column (Lisenoke Scientific Instruments (Shanghai) Co., Ltd., Shanghai, China) maintained at 45 °C, with the RID detector temperature set at 40 °C. Ultrapure water was used as the mobile phase at a flow rate of 1.0 mL/min.

The homogeneity of the purified polysaccharides was evaluated through chromatographic profiling using an HPLC data Origin 21 (OriginLab Corporation, Northampton, MA, USA). A calibration curve was generated with a series of dextran standards of known molecular weights (5 kDa, 12 kDa, 25 kDa, 50 kDa, 410 kDa, and 670 kDa). The molecular weights and uniformity of AOF1, AOF2, and AOF3 were then calculated based on the corresponding standard calibration curve.

#### 4.2.3. Ultraviolet Spectroscopy Analysis of AOFs

The UV spectroscopy of AOF1–3 (0.1 mg/mL) was scanned in the wavelength range of 190–700 nm using the Shimadzu UV-2700 UV-Vis spectrophotometer (Shanghai Jinghua Technology Instrument Co., Ltd., Shanghai, China). 

### 4.3. In Vitro Antioxidant Activity of AOFs

#### 4.3.1. DPPH Radical Scavenging Activi

The 2,2-diphenyl-1-picrylhydrazyl (DPPH) radical scavenging activity of AOF1, AOF2, and AOF3 was evaluated with minor modifications to a previously reported method [86]. Briefly, 100 μL of each polysaccharide sample at varying concentrations (0.4, 0.8, 1.2, 1.6, and 2.0 mg/mL) was mixed with 100 μL of 0.1 mmol/L DPPH ethanol solution. The mixtures were incubated in the dark at room temperature for 30 min, after which the absorbance was measured at 517 nm using a microplate reader (Hangzhou Aosheng Instrument Co., Ltd. Hangzhou, China). Vitamin C served as positive control. The DPPH radical scavenging activity was calculated using the following equation:

DPPH radical scavenging activity (%) = (A_2_ − (A_1_ − A_0_))/A_2_ × 100% (1)

where A_0_ represents the absorbance of the sample mixed with ethanol, A_1_ represents the absorbance of the sample mixed with DPPH ethanol solution, and A_2_ represents the absorbance of the control (DPPH ethanol mixed with distilled water).

#### 4.3.2. ABTS Radical Scavenging Activity

The 2,2′-azino-bis(3-ethylbenzothiazoline-6-sulfonic acid) (ABTS) radical scavenging activity of AOF1, AOF2, and AOF3 was determined with slight modifications to the method described by [40]. After incubation in the dark for 16 h, the ABTS radical solution was diluted with absolute ethanol to obtain an absorbance of 0.70 ± 0.02 at 734 nm. Subsequently, 100 µL of each sample solution (AOF1, AOF2, and AOF3) at concentrations of 0.4, 0.8, 1.2, 1.6, and 2.0 mg/mL was mixed with 100 µL of the diluted ABTS solution. The mixture was then incubated in the dark for 30 min at room temperature. The absorbance was measured at 734 nm, with vitamin C serving as the positive control. The radical scavenging activity was calculated using the following formula:

ABTS radical scavenging activity (%) = (A_2_ − (A_1_ − A_0_))/A_2_ × 100% (2)

where A_0_ is the absorbance of the sample with anhydrous ethanol, A_1_ is the absorbance of the sample with the diluted ABTS solution, and A_2_ is the absorbance of the water with the diluted ABTS solution.

#### 4.3.3. Hydroxyl Radical Scavenging Activity

The hydroxyl radical scavenging activity was evaluated according to previously reported methods, with minor modifications [87]. To assess hydroxyl radical scavenging activity, 50 μL of each sample (AOF1, AOF2, and AOF3) at concentrations of 0.4, 0.8, 1.2, 1.6, and 2.0 mg/mL was mixed with 50 μL of 2.25 mmol/L FeSO_4_ solution and 50 μL of 9 mmol/L salicylic acid in ethanol. Subsequently, 50 μL of 8.8 mmol/L hydrogen peroxide (H_2_O_2_) was added, and the mixture was incubated at 37 °C for 30 min. Absorbance was measured at 510 nm. In the blank group, distilled water replaced the sample, while in the control group, H_2_O_2_ was substituted with water. Vitamin C was used as a positive control. The hydroxyl radical scavenging activity was calculated using the following formula:

Hydroxyl radical scavenging activity (%) = (A_2_ − (A_1_ − A_0_))/A_2_ × 100% (3)

where A_0_ is the absorbance of the control group, A_1_ is the absorbance of the sample group, and A_2_ is the absorbance of the blank group.

### 4.4. Anti-Aging Activity of AOFs on C. elegans

#### 4.4.1. Cultivation and Synchronization of *C. elegans*

The wild-type *C. elegans* strain N2 was obtained from Titan Technology Co., Ltd. (Shanghai, China). All worms were maintained on nematode growth medium (NGM) plates and fed with *Escherichia coli* OP50 at 20 °C. To obtain synchronized populations, worms were selected during their peak reproductive period and transferred onto fresh NGM plates, followed by incubation at 20 °C. For synchronization, adult worms were collected, washed with M9 buffer, and lysed using a bleach solution (NaOH:NaClO:H_2_O = 1:1:8) to isolate eggs. The resulting embryos were subsequently incubated under standard culture conditions to obtain synchronized *C. elegans* populations for experimental assays.

#### 4.4.2. Determination of Lifespan

Wild-type *C. elegans* N2 were maintained on standard nematode growth medium (NGM) seeded with *E. coli* OP50 at 20 °C. Following synchronization, L4-stage worms were transferred to NGM plates containing AOFs at concentrations of 0.25, 0.5, and 1.0 mg/mL, as well as control plates without polysaccharides. To prevent progeny production, 5-fluoro-2′-deoxyuridine (FUDR, 70 mM) was added to the medium. Lifespan assays were conducted in accordance with the methods described by [88]. The day of transfer to treatment plates was designated as experimental 0 day. Worms were transferred to fresh plates every other day, and the number of surviving individuals was recorded daily until all worms had died. Each experiment was performed in triplicate, with 30 worms randomly selected per treatment group.

#### 4.4.3. Determination of Reproduction

Reproductive capacity of *C. elegans* was assessed following the method described by [88]. Briefly, L4-stage worms were transferred to FUDR-free NGM plates and cultured under standard conditions. For each experimental replicate, 10 wild-type N2 worms (one worm per plate) were individually transferred to fresh plates daily throughout the reproductive period. The plates containing eggs were incubated at 20 °C for 24 h to allow hatching. The number of offspring produced per worm was recorded at the L4 stage to confirm successful hatching. Each experiment was performed in triplicate, with 10 worms randomly selected per replicate.

#### 4.4.4. Determination of Motion

Locomotor activity of *C. elegans* was assessed by quantifying body bends, following a modified protocol based on previous methods [89]. Briefly, L4-stage worms were treated for 2 or 7 days as described in the lifespan assay. Adult worms were then transferred to fresh NGM plates, and the number of body bends defined as a complete change in the direction of the midbody was counted over a 30 s interval. Each experiment was conducted in triplicate, with 20 worms randomly selected per replicate to ensure statistical reliability.

#### 4.4.5. Determination of Lipofuscin Content

The L4-stage *C. elegans* were cultured on NGM plates with or without polysaccharide supplementation for 7 days. Following incubation, worms were anesthetized and observed under a fluorescence microscope to assess fluorescence intensity. Both body size and fluorescence intensity were quantitatively analyzed using ImageJ software (version 1.53). Each experiment was performed in triplicate, with 30 worms randomly selected per treatment group to ensure reproducibility and statistical robustness.

#### 4.4.6. Effects of AOFs on Stress Resistance of *C. elegans*

Stress tolerance of *C. elegans* was evaluated through heat, ultraviolet (UV) irradiation, and paraquat (PQ)-induced oxidative stress assays. For heat stress, synchronized L4-stage worms were transferred to NGM plates containing 70 mM 5-fluoro-2′-deoxyuracil (FUDR) to inhibit reproduction and treated with AOFs at 0.25, 0.5, 1.0, and 1.5 mg/mL for 6 days, followed by exposure to 37 °C until all worms died, with survival recorded hourly. UV stress tolerance was determined according to the described method [49], in which L4 worms were treated with polysaccharides (0.25, 0.5, 1.0 mg/mL) for 6 days on FUDR-containing plates and then exposed to a 254 nm UV lamp, and the number of living and dead worms was monitored daily until complete mortality. PQ-induced oxidative stress was assessed following previously reported methods [89,90] with slight modifications [91]. The worms cultured for 5 days with or without polysaccharides were incubated in 1 mL M9 buffer containing 125 mM paraquat for 2 h, washed three times with M9 buffer, and transferred to NGM plates seeded with *E. coli* OP50. After 24 h incubation at 20 °C, viable worms were counted. All experiments were performed in triplicate, with 30 worms randomly selected per replicate.

#### 4.4.7. Determination of ROS Content

The L4-stage N2 *C. elegans* were cultured on NGM plates with AOFs at concentrations of 0.25, 0.5, and 1.0 mg/mL, as well as control plates without polysaccharides, for 7 days. Following treatment, worms were washed with M9 buffer, and 100 μL of 200 μM H_2_DCF-DA was added. Worms were incubated at 22 °C for 2 h, after which excess H_2_DCF-DA was removed by washing with buffer, and the worms were anesthetized. Fluorescence images were captured using a fluorescence microscope (CKX-53 Yijingtong Optical Technology (Shanghai) Co., Ltd., Shanghai, China), and fluorescence intensity was quantified using ImageJ software. All experiments were performed in triplicate, with 30 randomly selected per treatment group to ensure reproducibility and statistical reliability.

#### 4.4.8. Determination of SOD, CAT Activity and MDA Levels

The L4-stage N2 *C. elegans* were transferred to NGM plates containing AOFs at concentrations of 0.25, 0.5, and 1.0 mg/mL, as well as control plates without polysaccharides. After 5 days of treatment, worms were washed with M9 buffer, and the collected sediment was resuspended in an appropriate volume of saline. The mixture was then homogenized and centrifuged to obtain the supernatant. The resulting extracts were used to determine superoxide dismutase (SOD) and catalase (CAT) activities, as well as malondialdehyde (MDA) levels, according to the instructions of commercial assay kits. All experiments were performed in triplicate to ensure reproducibility.

#### 4.4.9. Quantitative Real-Time PCR (qPCR)

Transfer the L4 stage N2 *C. elegans* to plates containing polysaccharides (0.25, 0.5, 1.0 mg/mL) or without polysaccharides. After 5 days of rearing, wash the *C. elegans* with M9 buffer to collect the sediment. Sample RNA was extracted from the tissue total RNA isolation kit. RNA was reverse-transcribed into cDNA and its relative expression was determined by real-time PCR. The primers are listed in Table S2.

#### 4.4.10. Prediction of Transcription Factor Binding Site

Based on the target species, promoter regions of the target genes hsp-16.2, sod-3, and ctl-1 were extracted from the NCBI database, specifically selecting sequences 2000 bp upstream of the transcription start site (TSS). Utilizing the Table Browser tool, the genome version and coordinates of the target genes were specified, and the sequences were exported in FASTA format. The retrieved promoter sequences were then submitted to the CISBP database to acquire binding motif information for the transcription factors DAF-16 and SKN-1. Promoter binding sites were predicted and screened using the Position Weight Matrix (PWM). Threshold parameters were set with a relative score threshold of ≥80% to balance the sensitivity and specificity of the predictions. A list of potential transcription factor binding sites within the promoter regions of the target genes was generated, encompassing site sequences, positions, strand orientations, and match scores.

### 4.5. Network Pharmacology and Molecular Docking Analysis to Explore the Anti-Aging Mechanism of AOFs

#### 4.5.1. Network Target Collection

Potential molecular targets of AOF1, AOF2, and AOF3 were predicted using an integrated in silico approach. Network pharmacology prediction was based on previously reported methods [32]. First, the chemical structures, oral bioavailability (OB), and drug-likeness (DL) of the constituent monosaccharides of each polysaccharide were retrieved from the TCMSP database (http://www.tcmip.cn/TCMIP/index.php/, accessed on 20 June 2025). Standard SMILES representations for these monosaccharides were obtained from the PubChem database (https://pubchem.ncbi.nlm.nih.gov/, accessed on 20 June 2025). Potential targets were then predicted using the Swiss Target Prediction database (http://www.swisstargetprediction.ch/, accessed on 20 June 2025) based on the 2D and 3D structural characteristics of known ligands. Target annotations were standardized using the UniProt database (https://www.uniprot.org/, accessed on 20 June 2025) to obtain corresponding gene names. After merging targets from all databases and removing duplicates, 1065, 494, and 657 gene targets were retained for AOF1, AOF2, and AOF3, respectively.

Aging-related targets were collected from multiple databases. Specifically, the GeneCards database (https://www.genecards.org/, accessed on 20 June 2025) was queried with a Z-score threshold of ≥1. Additional targets were retrieved from the OMIM database (https://www.omim.org, accessed on 20 June 2025) and DrugBank Online (https://go.drugbank.com/, accessed on 20 June 2025), selecting entries restricted to *Homo sapiens*. All target names were standardized and converted to gene symbols using UniProt. After merging datasets and removing duplicates, 1221 aging-associated gene targets were identified. This integrative approach enabled a comprehensive mapping of potential interactions between AOF and aging-related molecular pathways.

#### 4.5.2. Protein–Protein Interaction (PPI) Analysis

The predicted targets of AOF1, AOF2, and AOF3 polysaccharides, along with aging-associated targets, were submitted to the Venny 2.1.0 database (https://bioinfogp.cnb.csic.es/tools/venny/, accessed on 20 June 2025) to construct Venn diagrams and identify intersecting targets potentially involved in anti-aging effects. The overlapping targets were subsequently imported into the STRING database (https://string-db.org/, accessed on 20 June 2025) for protein–protein interaction (PPI) analysis, with *Homo sapiens* selected as the genetic background. The resulting PPI network files were then loaded into Cytoscape 3.10.3, and the Centiscape 2.2 plug-in was employed to calculate the degree values of nodes, with the top 10 genes selected as key targets for further analysis.

#### 4.5.3. GO and KEGG Enrichment Analysis

Gene Ontology (GO) enrichment and Kyoto Encyclopedia of Genes and Genomes (KEGG) pathway analyses were performed for the intersecting targets of AOF1, AOF2, and AOF3 polysaccharides and aging-related genes using the DAVID bioinformatics tool (https://davidbioinformatics.nih.gov/, accessed on 20 June 2025). Enrichment results were collated based on *p*-values and categorized into three GO domains: cellular component (CC), molecular function (MF), and biological process (BP). The top 10 enriched terms from GO analysis were visualized using a bubble plot, while the top 10 KEGG pathways were represented with a bar graph to illustrate key signaling pathways potentially involved in the anti-aging effects of AOF.

#### 4.5.4. Homology Modeling and Molecular Docking

Homology modeling of key anti-aging target proteins (AKT1, INS, SRC, and STAT3), identified through network pharmacology and retrieved from the UniProt database, was performed using the SWISS-MODEL platform to obtain the structural models with the highest sequence identity [92]. The quality and reliability of the modeled structures were subsequently assessed using Ramachandran plots. Operations such as the removal of water molecules and proto-ligands were conducted on the 4 target protein receptors using PyMOL 2.3.0. Small-molecule ligands, including arabinose, galactose, galacturonic acid, glucose and metformin (key monosaccharides in high abundance for significantly strong antioxidant efficacy and positive control) [93], corresponding to the monosaccharide composition of AOFs, were subjected to molecular docking with the key target proteins using PyRx (version 0.8) software. The docking parameters were set to: The center coordinates (unit: angstrom) of the proteins are (X: 14.08, Y: −13.80, Z: −13.59), (X: 10.56, Y: −0.63, Z: −2.18), (X: −3.48, Y: 38.37, Z: 38.20) and (X: −43.91, Y: 67.31, Z: 130.48), respectively. The dimensions (unit: angstrom) of the proteins are (X: 50.64, Y: 65.71, Z: 66.27), (X: 61.91, Y: 33.56, Z: 56.41), (X: 87.33, Y: 76.37, Z: 66.54) and (X: 172.02, Y: 67.31, Z: 130.48), respectively. The docking algorithm was a Lamarckian genetic algorithm, and the docking mode was semi-flexible docking, with the exhaustiveness set to 8, and the maximum number of conformations output was set to 8. The maximum number of conformations output was set to 9. Docking analyses were conducted to evaluate binding affinities and identify potential binding sites, providing mechanistic insights into the interactions between AOF-derived monosaccharides and anti-aging target proteins.

### 4.6. Statistical Analysis

Statistical analyses were performed using GraphPad Prism 10 (GraphPad Software, San Diego, CA, USA). Comparisons between the control and polysaccharide-treated groups were conducted using one-way analysis of variance (ANOVA). Survival data were analyzed using the Kaplan–Meier method in SPSS 20.0 (IBM Corp., Armonk, NY, USA). All results are presented as mean ± standard deviation (mean ± SD), and differences were considered statistically significant at *p* < 0.05.

## 5. Conclusions

This study systematically elucidated the anti-aging potential of AOF components both in vivo and at the molecular level, revealing the underlying mechanisms of their biological activity. The results demonstrated that AOF1, AOF2, and AOF3 each exhibit significant antioxidant and anti-aging effects. Mechanistically, the AOF components were found to enhance resistance to oxidative and heat stress in *C. elegans* by activating the insulin/insulin-like growth factor signaling (IIS) pathway, upregulating the activities of key antioxidant enzymes such as superoxide dismutase (SOD) and catalase (CAT), and reducing lipid peroxidation levels. Through these coordinated regulatory effects, AOFs effectively delayed senescence and significantly prolonged the healthy lifespan of *C. elegans*.

Network pharmacology analysis further revealed that the anti-aging effects of AOF1, AOF2, and AOF3 involve complex multi-target and multi-pathway interactions, suggesting that their efficacy arises from synergistic regulatory mechanisms rather than single-pathway modulation. This system-level insight provides a robust theoretical foundation for the further functional and pharmaceutical development of AOFs. Moreover, molecular docking results confirmed that all critical AOF monosaccharide components and metformin formed stable receptor–ligand complexes with key anti-aging targets (ATK1, INS, SRC, and STAT3) through extensive hydrogen bonding and van der Waals interactions, especially the galactose and galacturonic acid1021 components, which exhibited stronger anti-aging potential than the positive control-drug. Such molecular interactions contribute to the potent anti-aging activities observed experimentally.

Additionally, correlation analyses indicated a close relationship between the antioxidant capacity of AOFs and their lifespan-extending effects, underscoring the pivotal role of oxidative stress modulation in their mechanism of action. Notably, among the three fractions, AOF1 and AOF3 demonstrated superior antioxidant and longevity-promoting activities compared to AOF2, highlighting their potential as promising candidates for development as natural antioxidants or functional food ingredients targeting age-related decline. Collectively, this study not only provides valuable mechanistic insights into the anti-aging properties of AOFs but also establishes a scientific basis for their future application in the formulation of green, natural anti-aging therapeutics and functional foods.

## Data Availability

The original contributions presented in this study are included in the article/Supplementary Material. Further inquiries can be directed to the corresponding authors.

## Supplementary Materials

The following supporting information can be downloaded at: https://www.mdpi.com/article/10.3390/molecules31101698/s1, Figure S1: Standard curve of molecular weight of dextran. Figure S2: The binding motif of DAF-16 (**A**), prediction of DAF-16 binding sites in the promoter regions of SOD-3, HSP-16.2 and CTL-1 (**B**), the binding motif of SKN-1 (**C**), prediction of SKN-1 binding sites in the promoter regions of SOD-3 and HSP-16.2 (**D**). Figure S3: After homology modeling, the Ramachandran plots were tested including AKT1 (**A**), INS (**B**), SRC (**C**) and STAT3 (**D**). Figure S4: Correlation analysis of lifespan, sport, stress resistance, antioxidant and anti-aging gene expression in vivo of *Caenorhabditis elegans*. Where * represents *p* < 0.05, ** represents *p* < 0.01, and *** represents *p* < 0.001. Figure S5: Molecular docking of the anti-aging key target protein (ATK1, INS, SRC, and STAT3) and the Anti-aging positive control (metformin). Amino acid residue fragments are depicted by sticks, hydrogen bonds are shown as yellow dotted lines in three-dimensional structures. Table S1: Extraction rate of AOF1–3 polysaccharide. Table S2: Primers for qPCR detection. Table S3: Molecular weight (Mw) of AOF1, AOF2 and AOF3. Table S4: A list of potential transcription factor binding sites within the promoter regions of the target genes, encompassing site sequences, positions, strand orientations, and match scores. Table S5: Binding energy and residues involved in hydrogen bonding and hydrophobic interactions for key target proteins (AKT1, INS, SRC, and STAT3) bonding to composition of AOP monosaccharides (arabinose, galactose, galacturonic acid, glucose) and positive control metformin.

## Abbreviations

The following abbreviations are used in this manuscript:

ROS	reactive oxygen species
GSH-Px	glutathione peroxidase
SOD	superoxide dismutase
CAT	catalase
AO	*Alpinia oxyphylla*
AOFs	*Alpinia oxyphylla* polysaccharides
MDA	malondialdehyde
DPPH	2,2-diphenyl-1-picrylhydrazyl
ABTS	2,2′-azino-bis(3-ethylbenzothiazoline-6-sulfonic acid)
FUDR	5-fluoro-2′-deoxyuridine
qPCR	quantitative real-time PCR
OB	oral bioavailability
DL	drug-likeness
GO	gene ontology
KEGG	Kyoto Encyclopedia of Genes and Genomes
CC	cellular component
MF	molecular function
BP	biological process
AKT1	AKT serine/threonine kinase 1
INS	insulin
SRC	SRC proto-oncogene, non-receptor tyrosine kinase
STAT3	signal transducer and activator of transcription 3
ANOVA	analysis of variance
DEAE-52	Diethyl aminoethyl cellulose-52
HPGPC	high-performance gel permeation chromatography
UV	ultraviolet
IIS	insulin/insulin-like growth factor signaling
DAF-16	Dauer Formation-16
FoxO	Forkhead box O
SOD-3	superoxide dismutase-3
CTL-1	catalase-1
SKN-1	skinhead-1
hsp-16.2	heat-shock protein-16.2
PPI	protein–protein interaction
PI3K	positive regulation of phosphatidylinositol 3-kinase
AKT	AKT serine/threonine kinase 1
MAPK	mitogen-activated protein kinase
EGFR	epidermal growth factor receptor
HIF-1	hypoxia-inducible factor-1
ATK1	AKT serine/threonine kinase 1
