# Supplementary material for: Mechanisms of Anti-Aging Effect of Alpinia oxyphylla Polysaccharides Mediated via IIS Pathway: Based on In Vivo Experiments, Network Pharmacology and Molecular Docking
Source: Molecules. 2026 May 17;31(10):1698. doi: 10.3390/molecules31101698 (PMC13209729; doi:10.3390/molecules31101698)
Supplement: Supplementary file 1 [file molecules-31-01698-s001.zip › molecules-4270255-supplementary.pdf]

Figures

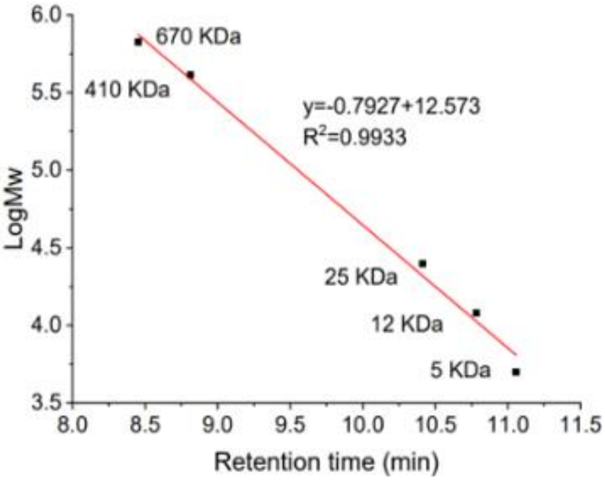

Figure S1. Standard curve of molecular weight of dextran.

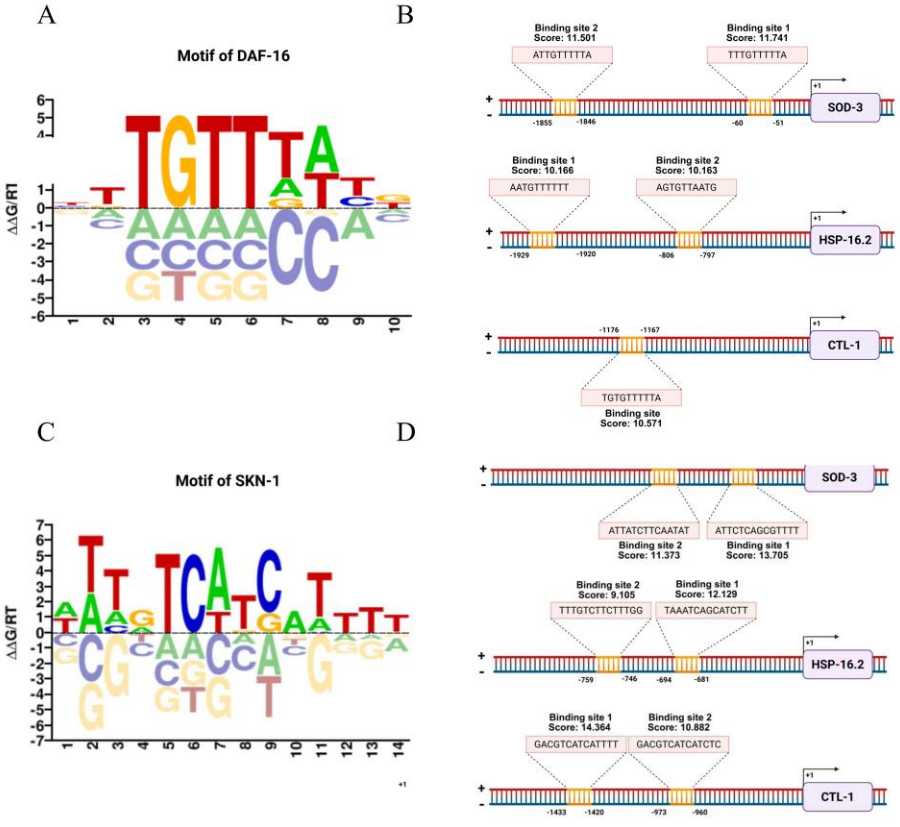

Figure S2. The binding motif of *DAF-16* (A), prediction of *DAF-16* binding sites in the promoter regions of *SOD-3*, *HSP-16.2* and *CTL-1* (B), the binding motif of *SKN-1* (C), prediction of *SKN-1* binding sites in the promoter regions of *SOD-3* and *HSP-16.2* (D).

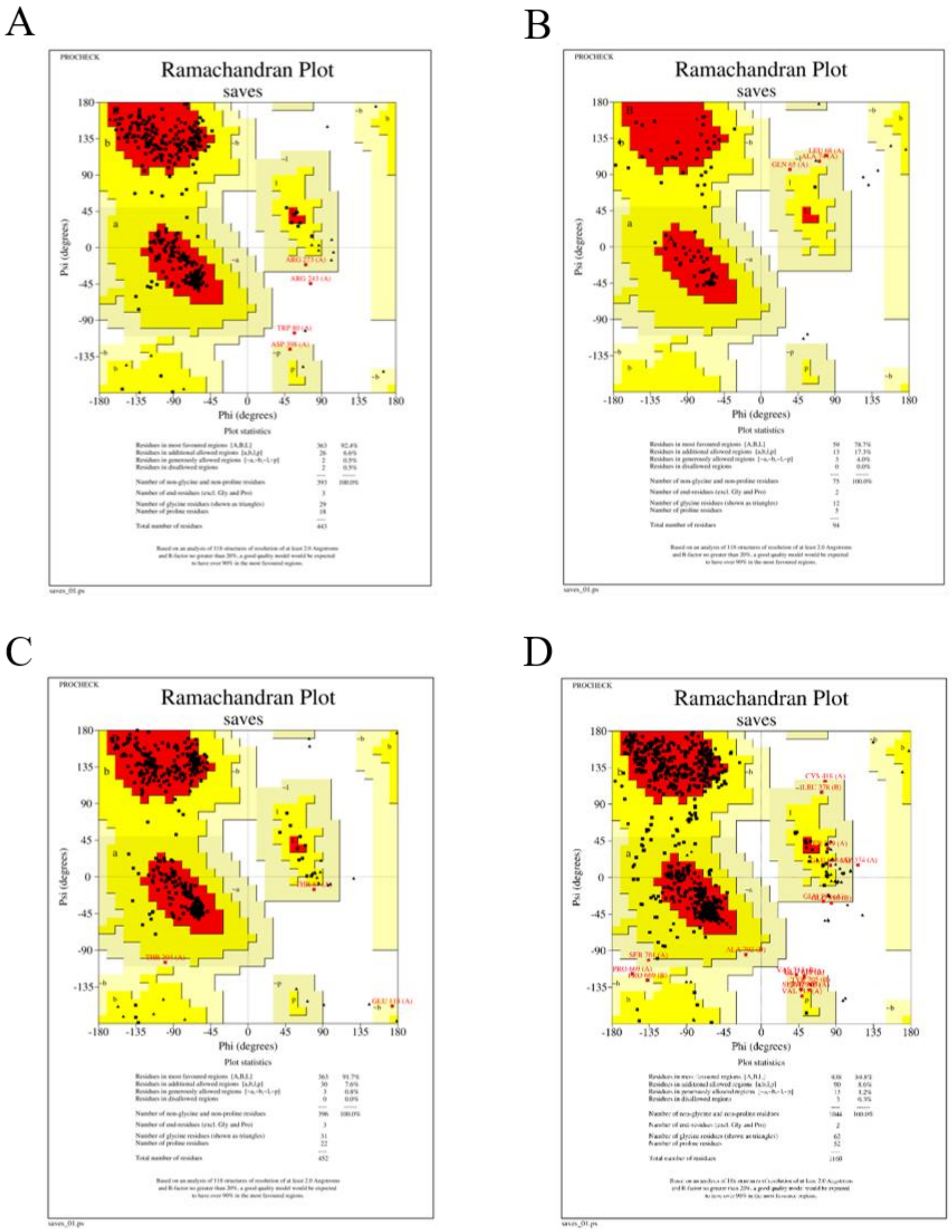

Figure S3. After homology modeling, the Ramachandran plots were tested including AKT1 (A), INS (B), SRC (C) and STAT3 (D).

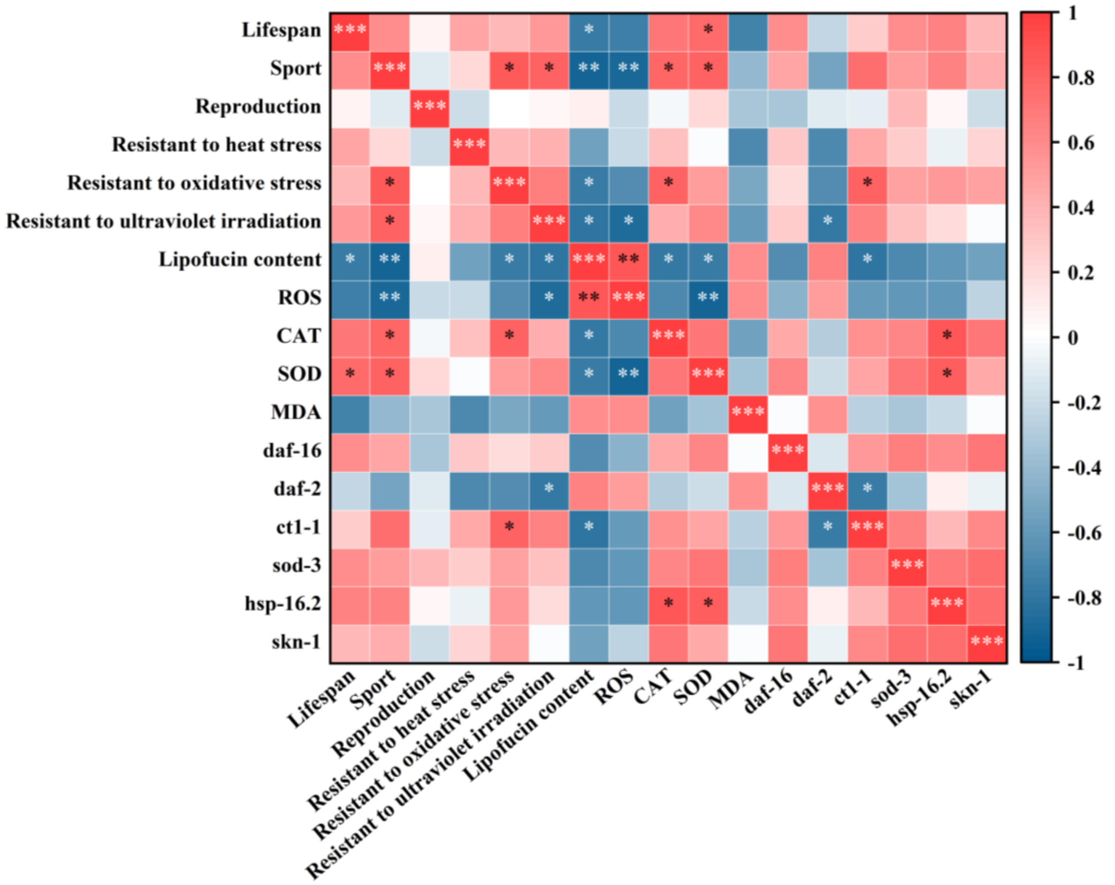

Figure S4. Correlation analysis of lifespan, sport, stress resistance, antioxidant and anti-aging gene expression in vivo of *Caenorhabditis elegans*. Where *represents $p<0.05$, **represents $p<0.01$, and ***represents $p<0.001$.

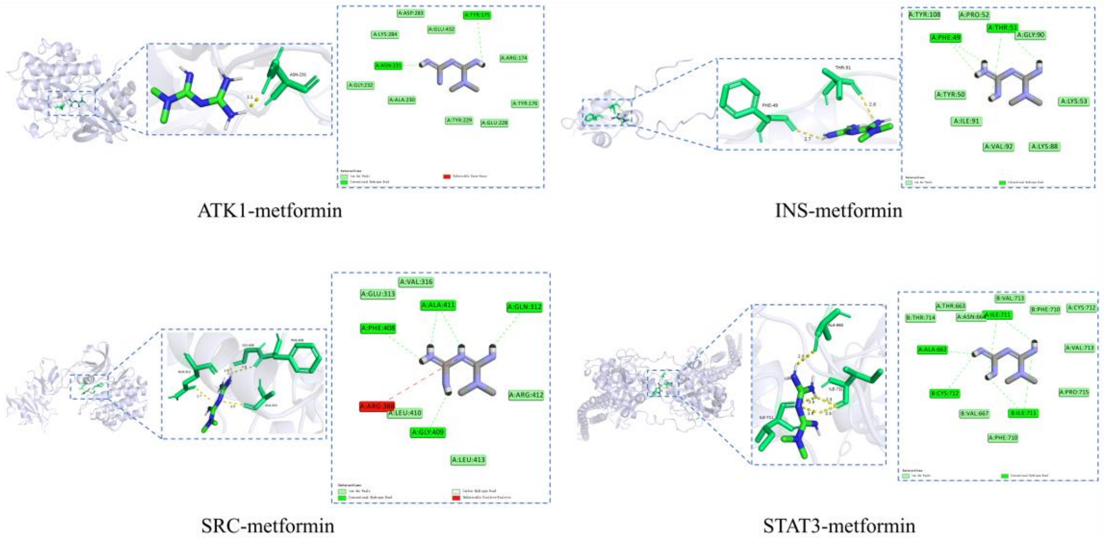

Figure S5. Molecular docking of the anti-aging key target protein (ATK1, INS, SRC, and STAT3) and the Anti-aging positive control (metformin). Amino acid residues fragment was depicted by sticks, hydrogen bonds were shown as yellow dotted lines in three-dimensional structures.

Tables

Table S1. Extraction rate of AOF1-3 polysaccharide

Sample name	For the extraction rate of crude polysaccharidez (%)	Extraction rate for sample (%)
AOF1	6.25	0.61
AOF2	16.79	1.64
AOF3	17.57	1.71

Table S2 Primers for qPCR detection

Genes	Forward primer	Reverse primer
act-1	GCTGATCGTATGCAGAAGGAAA	GTTGGAAGGTGGAGAGGGAAG
daf-16	CCAGACGGAAGGCTTAAACT	ATTCGCATGAAACGAGAATG
sod-3	CCAACCAGCGCTGAAATTCAATGG	GGAACCGAAGTCGCGCTTAATAGT
skn-1	AGTGTCGGCGTTCCAGATTTC	GTCGACGAATTGCGAATCA
daf-2	CGGTGCGAAGAGAGGATATT	TACAGAGGTCGCCGTTACTG
ctl-1	TCCTACACGGACACGCATTAC	CGGAAACTGTTCGGGAAGTAA
shp-16.2	CGCTATCAATCCAAGGAGAAC	GAAGCAACTGCACCAACATC

Table S3 Molecular weight (Mw) of AOF1, AOF2 and AOF3

Items	AOF1	AOF2	AOF3
Retention time (min)	6.326	6.946	6.554
Mw (kDa)	36172.0	11665.0	24298.0

Table S4 A list of potential transcription factor binding sites within the promoter regions of the target genes, encompassing site sequences, positions, strand orientations, and match scores.

Gene	TF	Binding Sequence	From	To	Direction	Score
SOD-3	DAF-16	TTTGTTTTTA	1940	1949	F	11.741
		ATTGTTTTTA	145	154	F	11.504
		TGTGTTAATT	14	23	F	10.275
		AATGTTTTTT	1431	1440	R	10.166
		TTTGTTAAGA	246	255	R	9.043
		ATTGTTTAAA	1882	1891	F	8.431
	SKN-1	ATTCTCAGCGTTTT	1855	1868	R	13.705
		ATTATCTTCAATAT	1828	1841	R	11.373
		TTTTTCACCTTTTT	905	918	F	11.193
		GTTTTCAAGATTTT	343	356	F	8.113
		AAAATCTTGAAGAT	1550	1563	R	8.11
HSP-16.2	DAF-16	AATGTTTTTT	71	80	F	10.166
		AGTGTTAATG	1194	1203	F	10.163
		AATGTTTAGA	751	760	R	8.72
		TTTGTTTAAA	102	111	F	8.668
	SKN-1	TAAATCAGCATCTT	1306	1319	F	12.129
		TTTGTCTTCTTTGG	1241	1254	F	9.105
CTL-1	DAF-16	TGTGTTTTTA	824	833	R	10.571
	SKN-1	GACGTCATCATTTT	567	580	F	14.364
		GACGTCATCATCTC	1027	1040	F	10.822

Table S5 Binding energy and residues involved in hydrogen bonding and hydrophobic interactions for key target proteins (AKT1, INS, SRC, and STAT3) bonding to composition of AOPs monosaccharides (arabinose, galactose, galacturonic acid, glucose) and positive control metformin.

Complex	Binding energy (kcal/mol)	Hydrophobic interactions	Hydrogen bonding
ATK1-arabinose	-4.9	\	Tyr229, Asn231, Lys284
ATK1-galactose	-5.0	\	Arg15, Thr87, Lys297
ATK1-galacturonic acid	-5.5	\	Asn54, Gln79, Thr81, Thr82, Val271, Tyr272, Asp292
ATK1-glucose	-5.1	\	Tyr175, Tyr229
ATK1-metformin	-5.0	\	Tyr175, Asn231
INS-arabinose	-4.0	\	Phe49, Thr51
INS-galactose	-4.0	\	Arg56, Gly84, Gln87, Lys88
INS-galacturonic acid	-4.0	\	Thr51
INS-glucose	-3.9	\	Phe49, Thr51, Lys88
INS-metformin	-4.1	\	Phe49, Thr51
SRC-arabinose	-4.2	\	Thr93, Gln147, Glu149
SRC-galactose	-4.9	\	Leu413, Glu415, Phe442
SRC-galacturonic acid	-4.8	\	Tyr93, Gln147, Glu150, Tyr152
SRC-glucose	-4.7	\	Met317, Thr341
SRC-metformin	-4.7	\	Gln312, Phe408, Gly409, Ala411
STAT3-arabinose	-4.6	\	Arg688, Pro689, Gln692
STAT3-galactose	-5.5	\	Asp369, Lys370, Asp371, Leu438, Thr440

STAT3-galacturonic acid	-5.5	\	Asp369, Asp371, Leu436, Leu438
STAT3-glucose	-5.1	\	Ser381, Lys383, Glu415, Asn425
STAT3-metformin	-5.2	\	Ala662, Ile711, Cys712
